# Epstein Barr virus-mediated transformation of B cells from XIAP-deficient patients leads to increased expression of the tumor suppressor CADM1

**DOI:** 10.1038/s41419-022-05337-z

**Published:** 2022-10-22

**Authors:** Christine Engelmann, Patrick Schuhmachers, Hana Zdimerova, Sanamjeet Virdi, Mathias Hauri-Hohl, Jana Pachlopnik Schmid, Adam Grundhoff, Rebecca A. Marsh, Wendy Wei-Lynn Wong, Christian Münz

**Affiliations:** 1 Viral Immunobiology, Institute of Experimental Immunology, University of Zürich, Zürich, Switzerland; 2 Virus Genomics, Heinrich Pette Institute, Hamburg, Germany; 3 Division of Immunology, University Children’s Hospital Zurich, Zurich, Switzerland; 4 Department of Pediatrics, University of Cincinnati, Cincinnati, OH USA; 5 Cell Death and Regulation of Inflammation, Institute of Experimental Immunology, University of Zürich, Zürich, Switzerland

**Keywords:** Primary immunodeficiency disorders, Gene regulation in immune cells

## Abstract

X-linked lymphoproliferative disease (XLP) is either caused by loss of the SLAM-associated protein (SAP; XLP-1) or the X-linked inhibitor of apoptosis (XIAP; XLP-2). In both instances, infection with the oncogenic human Epstein Barr virus (EBV) leads to pathology, but EBV-associated lymphomas only emerge in XLP-1 patients. Therefore, we investigated the role of XIAP during B cell transformation by EBV. Using humanized mice, IAP inhibition in EBV-infected mice led to a loss of B cells and a tendency to lower viral titers and lymphomagenesis. Loss of memory B cells was also observed in four newly described patients with XIAP deficiency. EBV was able to transform their B cells into lymphoblastoid cell lines (LCLs) with similar growth characteristics to patient mothers’ LCLs in vitro and in vivo. Gene expression analysis revealed modest elevated lytic EBV gene transcription as well as the expression of the tumor suppressor cell adhesion molecule 1 (CADM1). CADM1 expression on EBV-infected B cells might therefore inhibit EBV-associated lymphomagenesis in patients and result in the absence of EBV-associated malignancies in XLP-2 patients.

## Introduction

XIAP-deficiency is a rare primary immunodeficiency disease also called X-linked lymphoproliferative disease 2 (XLP-2) that affects about 1–2 in a million males [1]. The disease was first characterized in 2006 by Rigaud et al. [2] in patients with characteristic symptoms of XLP, including hemophagocytic lymphohistiocytosis (HLH), hypogammaglobulinemia and cytopenia, but lacking mutations in the SLAM-associated protein (SAP) gene, which at the time was the known genetic defect to cause XLP (XLP-1). Genotype analysis of the X chromosome led to the discovery of genetic mutations in the *XIAP* gene, encoding the X-linked inhibitor of apoptosis (XIAP). In contrast to XLP-1 patients, which are exquisitely susceptible to pathology driven by oncogenic Epstein Barr virus (EBV) infection, EBV is not the only driver of disease in XLP-2. Nevertheless, HLH is often triggered by EBV infection, but in contrast to XLP-1 EBV-driven lymphomas have not been observed in XLP-2 [3].

XIAP is a ubiquitously expressed protein and a member of the inhibitor of apoptosis (IAP) family, which also includes cellular IAP1 (cIAP1), cellular IAP2 (cIAP2), melanoma IAP (ML-IAP), neuronal apoptosis inhibitory protein (NAIP), survivin, Appolon and IAP-like protein 2 (ILP2) [4]. Members of the IAP family are characterized by the presence of one to three baculoviral IAP repeat (BIR) domains, which confer their ability to interact with specific binding partners [5]. As their name suggests, IAPs are involved in the regulation of the apoptotic pathway either solely by binding to its key components and/or by ubiquinating these for proteasomal degradation. Indeed, XIAP can directly bind to and inhibit the executioner caspases 3 and 7 with its BIR1-BIR2 linker domain [6] and to the initiator caspase 9 with its BIR3 domain [7]. Besides modulation of apoptosis, IAPs are involved in a plethora of other signaling pathways, including NF-κB and MAPK pathways [8, 9], autophagy [10–12], as well as DNA damage, Wnt signaling, cell motility and migration [13, 14]. Therefore, XIAP might support EBV-associated lymphomagenesis via a variety of mechanisms.

In this study, we aimed to shed light on the role of XIAP in lymphomagenesis during primary EBV infection and in established immortalized lymphoblastoid cell lines (LCLs) from XIAP-deficient patients. Pharmacological inhibition of IAPs using SMAC-mimetics in a humanized mouse model of EBV infection led to a reduction in circulating B cells, a phenotype primarily found in memory B cells of XIAP-patients and slightly reduced viral loads and tumor development. RNA sequencing of established LCLs from patients and their mothers revealed the induction of the tumor suppressor cellular adhesion molecule 1 (CADM1) in patient-derived cells. Even though CADM1 was not required for the growth of LCLs in vitro, it might contribute to the absence of EBV-associated lymphomas in XLP-2 patients.

## Results

### SMAC-mimetic treatment leads to a decrease of B cells in humanized mice

**Fig. 1:**
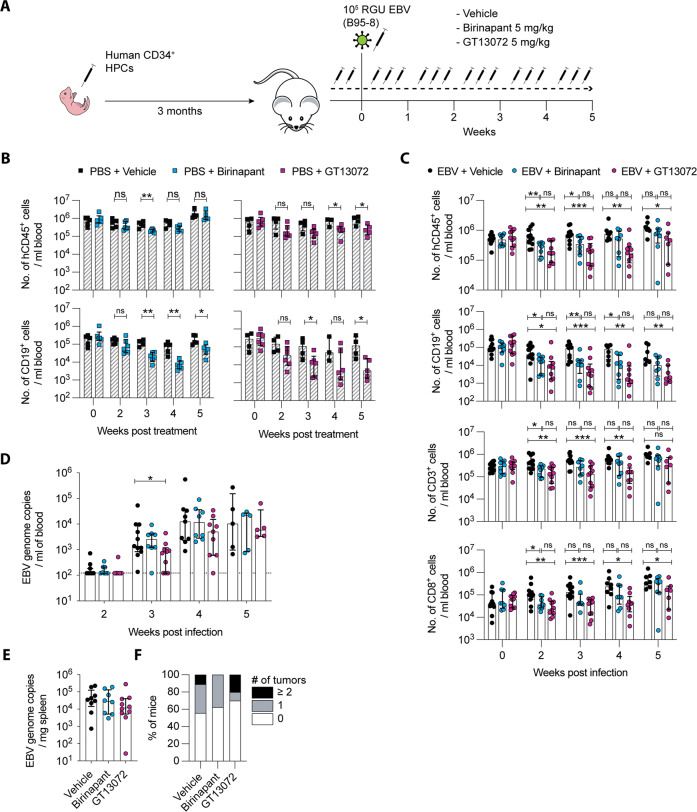
SMAC-mimetic treatment leads to reduction in lymphoid cells. **A** Experimental procedure of SMAC-mimetic treatment in humanized mice. Male and female mice were distributed into vehicle or treatment groups with the SMAC-mimetics Birinapant or GT13072, with or without EBV infection. Compounds were injected intraperitoneally three times a week until the end of the experiment, starting with two injections prior to infection. **B** Total numbers of human CD45^+^ leukocytes and CD19^+^ B cells during the treatment with Birinapant (blue, *n* = 6), GT13072 (purple, *n* = 6) and vehicle (black, *n* = 5 and 4, respectively) of humanized mice. The data originate from mice reconstituted with hCD34^+^ cells derived from 1 donor (Birinapant) or 2 donors (GT13072). **C** Total numbers of human CD45^+^ cells, CD19^+^ B cells, CD3^+^ and CD8^+^ T cells during EBV infection under SMAC-mimetic treatment with Birinapant (blue, *n* = 8), GT13072 (purple, *n* = 10) or vehicle (black, *n* = 11). The data originate from mice reconstituted with hCD34^+^ cells derived from three different donors. **D** EBV genome copies per ml of blood from mice treated with Birinapant (blue, *n* = 8), GT13072 (purple, *n* = 10), or vehicle (black, *n* = 11) during the course of the experiment as determined by qPCR detecting the BamHI-W fragment of EBV. **E** EBV genome copies per mg spleen. Data include all mice that survived at least until week 4 (Birinapant, *n* = 8; GT13072, *n* = 10; vehicle, *n* = 9). **F** Tumor incidence in SMAC-mimetic-treated or untreated mice. Number of macroscopically visible tumors in organs or in the peritoneum were determined at the end of the experiment (Birinapant, *n* = 8; GT13072, *n* = 10; vehicle, *n* = 9). **B**–**F** Each dot represents one animal (median + IQR). *p < 0.05, **p < 0.01, ***p < 0.001, significant comparisons are indicated and significance was determined using Mann–Whitney *U* test.

SMAC-mimetics are small molecules being evaluated in clinical trials, either alone or in combination with chemotherapeutics to treat a wide range of cancer types [15]. SMAC/Diablo is a naturally occurring mitochondria-derived protein that interacts with IAPs to antagonize their anti-apoptotic function [16, 17]. SMAC-mimetics bind to cIAPs and trigger their auto-ubiquitylation, targeting them for proteasomal degradation and prevent XIAP from binding and thereby inhibiting caspase activation [4]. IAPs are often overexpressed in cancer [18] and correlate with worse outcomes. In this study, we aimed to investigate the role of XIAP loss in primary EBV infection. To this end, we made use of the second-generation bivalent SMAC-mimetic Birinapant, targeting cIAP1 and cIAP2 [19], and the bivalent GT13072, which targets cIAPs and blocks XIAP function [20]. With this differential effect of the used SMAC-mimetics on cIAPs and XIAP inhibition, we aimed to pharmacologically interrogate cIAPs with and without XIAP during primary EBV infection of NOD-*scid* γ_c_^−/−^ (NSG) mice with reconstituted human immune system components (humanized mice; Fig. 1A). This humanized mouse model is an established model to study primary EBV infection in vivo, recapitulating some of the hallmarks of infectious mononucleosis (IM), including an up to tenfold increase in CD8^+^ T cells [21]. Additionally, 20–30% of these mice develop lymphoproliferative lesions, enabling the study of the tumorigenic potential of EBV [22]. Reduction of cIAP1 after SMAC-mimetic treatment was verified by Western blot (Fig. S1B, D). The efficacy of XIAP-blockade by GT13072 was shown by inhibiting the NOD2 pathway in PBMC-derived monocytes [23]. The addition of GT13072 was able to reduce TNF-α secretion in L18-MDP stimulated monocytes, whereas Birinapant was much less able to do so at the same concentration (Fig. S1E). IAP depletion in non-infected humanized mice led to a moderate reduction of human CD45^+^ cells, resulting predominantly from a decrease of B cells in the peripheral blood (Fig. 1B), whereas T and NK cells remained largely unaffected (Fig. S1A, C). This phenotype is reminiscent of patients treated with Birinapant [24]. EBV infection in SMAC-mimetic treated mice resulted in a more pronounced reduction of B and T cells compared to vehicle mice (Fig. 1C and Fig. S1F). Additionally, GT13072–treatment induced a stronger depletion than Birinapant, suggesting a role for XIAP in B and T cell maintenance in humanized mice. In line with this, T cells from XIAP patients are described to present with increased activation induced cell death, with B cells not yet characterized. B cells in humanized mice are predominantly immature and rarely undergo class-switching due to poor development of secondary lymphoid tissues [25]. Thus, we measured total IgM serum levels at termination to determine whether plasma B cells would preferentially be depleted by SMAC-mimetic treatment. Slightly lower total IgM titers, albeit not significant, were determined in GT13072-treated mice (Fig. S1H). Monitoring memory B cells during EBV infection and SMAC-mimetic treatment is masked due to the upregulation of CD27 by EBV in B cells (Fig. S1I). With B cells being significantly reduced in treated mice, EBV viral loads were also lower at week 3 post infection in peripheral blood of GT13072-treated mice (Fig. 1D). However, no difference was detected in splenic tissues or serum at sacrifice (Fig. 1E; Fig. S1G). Moreover, fewer mice developed tumors in the GT13072-treated mice (30%) compared to vehicle mice (44.4%) albeit this difference was not statistically significant (Fig. 1F). These data suggest that SMAC-mimetics compromise the human B cell compartment in humanized mice along with a tendency towards lower EBV infection. Importantly, the compromised immune response through XIAP-inhibition did not further aggravate tumorigenesis, suggesting that loss of XIAP does not lead to tumor formation, as seen in XIAP patients.

### Reduction of circulating memory B cells in XIAP-deficient patients

**Fig. 2:**
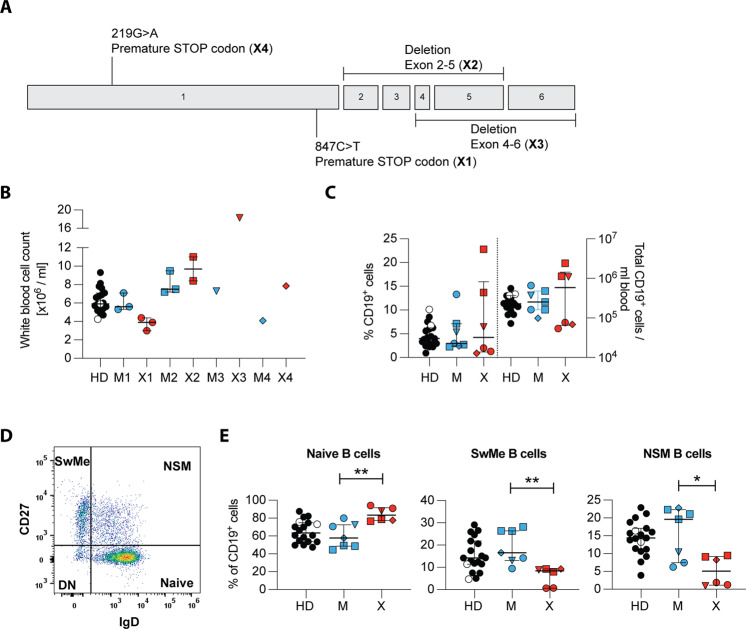
Phenotypic characterization of peripheral blood lymphocytes in XIAP-deficient patients and their mothers. **A** Graphical depiction of mutations in XIAP patients described in this study. Numbered boxes represent the 6 coding exons of XIAP. **B**–**E** Phenotypic characterization in healthy donors (HD, black), including 2 children aged 6 and 10 years (white circles), female carriers (M, blue), and XIAP patients (X, red). Each family was assigned a distinct symbol. **B** White blood cell count of whole blood determined on the day of blood draw. Each blood donation is shown, number of donations per family were either one, two, or three. **C** Percentage and total cell count of peripheral CD19^+^ B cells in HD (*n* = 19), M (*n* = 7) and X (*n* = 6). **D** Representative flow cytometric plot of naïve, switched-memory (SwMe), non-switched memory (NSM) and double negative (DN) B cells as determined by IgD and CD27 expression. **E** Frequencies of naïve, SwMe and NSM B cell subsets within all CD19^+^ cells in HD, M, and X. Error bars represent median + IQR. Statistical significance was calculated using a Mann–Whitney *U* test, *p < 0.05; **p < 0.01.

Next we investigated the immune cell composition in the peripheral blood of four unrelated XIAP patients and their respective mothers, carriers of the mutation. Patient characteristics are summarized in Table S1. Each patient presented with a different mutation in the *XIAP* gene, that led to the complete absence of the protein. Premature STOP codons or deletions of several exons were detected (Fig. 2A). We assessed the total white blood cell count of each donor together with immunophenotyping to monitor immune cell cytopenia, a frequent symptom described in these patients [2, 26, 27]. Patient 1 (X1) presented on three different time points with a mean total leukocyte count of 3.8 × 10^6^/ml, the lower end of the normal range. Patient 3 (X3) was assessed at one time point and showed a massive expansion of total leukocyte count, most likely due to chronic inflammation caused by IBD symptoms. On the other hand, patients 2 (X2) and 4 (X4) were in the normal range (Fig. 2B). Distribution of different immune cell subsets was assessed by flow cytometry. In general, each patient presented with a distinct immunophenotype (Fig. 2C and Fig. S2). Patient 1 and 4 presented with a reduced frequency and total number of CD19^+^ B cells (Fig. 2C). Additionally, we differentiated B cell subsets into naïve, switched-memory, non-switched memory and double negative B cells according to the expression of the markers CD27 and IgD (Fig. 2D). All described patients displayed significantly increased naïve and reduced memory B cell pools compared to their healthy mothers (Fig. 2E). To exclude that lower cIAP1 or cIAP2 expression levels in memory B cells could render these more susceptible to depletion after XIAP loss we assessed their respective expression by Western blot in flow cytometric-sorted switched-memory and naive B cells of healthy donors. No difference in cIAP1 or cIAP2 levels were detected in both B cell subsets (Fig. 2SC). Therefore, in line with IAP inhibition in humanized mice, XIAP deficiency seems to affect the human B cell compartment in patients. More specifically, in XIAP patients memory B cells are depleted probably as a result of a general requirement for IAPs in B cells as revealed by our pharmacological inhibition in humanized mice.

### XIAP-deficiency does not influence the proliferation and cell death rate of EBV-transformed primary B cells

**Fig. 3:**
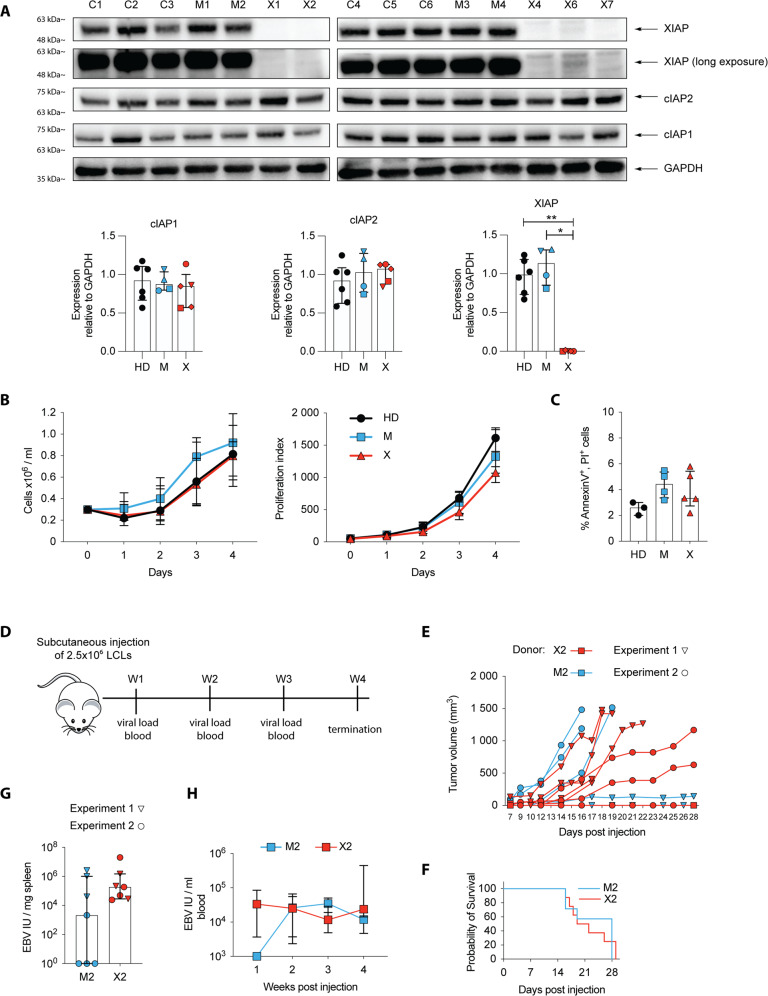
Loss of XIAP in LCLs does not affect their proliferation, spontaneous apoptosis or tumorigenesis. **A** Western blot analysis of XIAP, cIAP1, and cIAP2 expression in LCLs derived from healthy donors (C), female carriers (M) and XIAP-patients (X). Quantification of cIAP1, cIAP2, and XIAP relative to GAPDH. **B** Total cell count as determined by trypan blue exclusion of LCL cell cultures over 4 days (Left). Proliferation rate as determined by the inverse of the median fluorescent intensity (MFI) of Cell Trace Violet ×10^6^ of each cell line (right). Cells were seeded at 0.3 × 10^5^ cells/ml after CTV staining and analyzed every day for total cell counts and CTV expression. **C** Frequency of apoptotic cells after 4 days in culture determined by AnnexinV/PI staining. **D** Experimental scheme of LCL transfer experiment. 2.5 × 10^6^ LCLs were subcutaneously injected into the right flank. Tumor growth was monitored once a palpable tumor was formed by calipering every second day and every day once the tumor reached a width of 1 cm. Mice were euthanized when the tumor reached a maximum width of 1.5 cm. **E** Tumor volume of individual mice during the course of the experiment. Tumor volume was calculated with the formula (π × length × width^2^)/6, with length representing the largest tumor diameter and width the perpendicular tumor diameter. **F** Kaplan–Meier survival curve of animals injected with either XIAP patient- or mother-derived LCLs. **G** EBV DNA loads in the spleen of mice. **H** EBV viral loads in the peripheral blood. **D**–**H** Data are from two independent experiments. XIAP patient LCL-injected mice, *n* = 7; mother LCL-injected mice, *n* = 7. **B**–**C**, **G,** **H** Data are presented as median + IQR. **A**, **C**, **G**, **H** Statistical significance was assessed with the Mann–Whitney *U* test, *p < 0.05; **p < 0.01.

To investigate if loss of XIAP plays a role in EBV-infected B cells, we isolated B cells of XIAP patient-derived PBMCs, their mothers, and healthy donor controls and infected them with the EBV B95-8 strain at a multiplicity of infection (MOI) of 0.1. XIAP loss did not interfere with EBV transformation of primary B cells and thus lymphoblastoid cell lines (LCLs) were generated from four mothers (M1, M2, M3, and M4) and from three patients (X1, X2, X4). In line with this, unchanged XIAP transcript levels during primary EBV infection of healthy human B cells in vitro suggest that EBV does not regulate XIAP expression (Fig. S3A) [28]. The expression levels of XIAP in control, mother and patient LCLs was confirmed by Western blot analysis. Additional XIAP patient-derived LCLs (X6, X7) provided by R. Marsh were analyzed (Fig. 3A). Long exposure shows residual expression of XIAP in LCLs derived from patient X6. Mother-derived LCLs showed similar XIAP expression levels as healthy donor controls. Additionally, the expression of cIAP1 and cIAP2 remained unaffected by XIAP loss, as shown by similar expression levels in all tested LCLs. As XIAP plays a major role in protecting cells from apoptosis, we assessed the growth rate and spontaneous cell death of the generated LCLs. However, the absence of XIAP did not impair proliferation of LCLs as shown by comparable growth rates of each cell line (Fig. 3B and Fig. S3B). Each LCL also contained similar frequencies of spontaneous cell death (Fig. 3C). The successful transformation of XIAP-deficient primary B cells by EBV in vitro and the similar growth rates and incidence of spontaneous apoptosis in established LCLs suggests that XIAP is not involved in these processes, underpinning the strong transformation capability of EBV.

XIAP is also shown to regulate cell migration [13, 14], therefore we assessed the migration capacity of LCLs in an in vivo model using NSG mice. We injected LCLs from patient 2 or mother 2 subcutaneously into the right flank of NSG mice and monitored tumor development and growth as well as subsequent spreading by measuring EBV viral loads in the spleen and peripheral blood (Fig. 3D). In most mice a palpable tumor formed after a week, which was then monitored until four weeks post injection or until the tumor reached a maximum length of 1.5 cm, which was the humane endpoint for euthanasia. Tumors from each donor grew fast or slow, depending on the experiment (Fig. 1E). Thus, mice had to be euthanized at a similar rate (Fig. 3F). Additionally, EBV viral loads in the spleen and blood did not differ between the two donors (Fig. 3G, H). Consequently, loss of XIAP also did not impair tumor formation and did not prevent LCLs from spreading via the circulation into the spleen. Thus, XIAP does not seem to be required for EBV induced B cell transformation in vitro and has no significant effect on tumor formation by the resulting LCLs in vivo.

### RNAseq analysis reveals increased expression of lytic EBV genes in XIAP-deficient LCLs

**Fig. 4:**
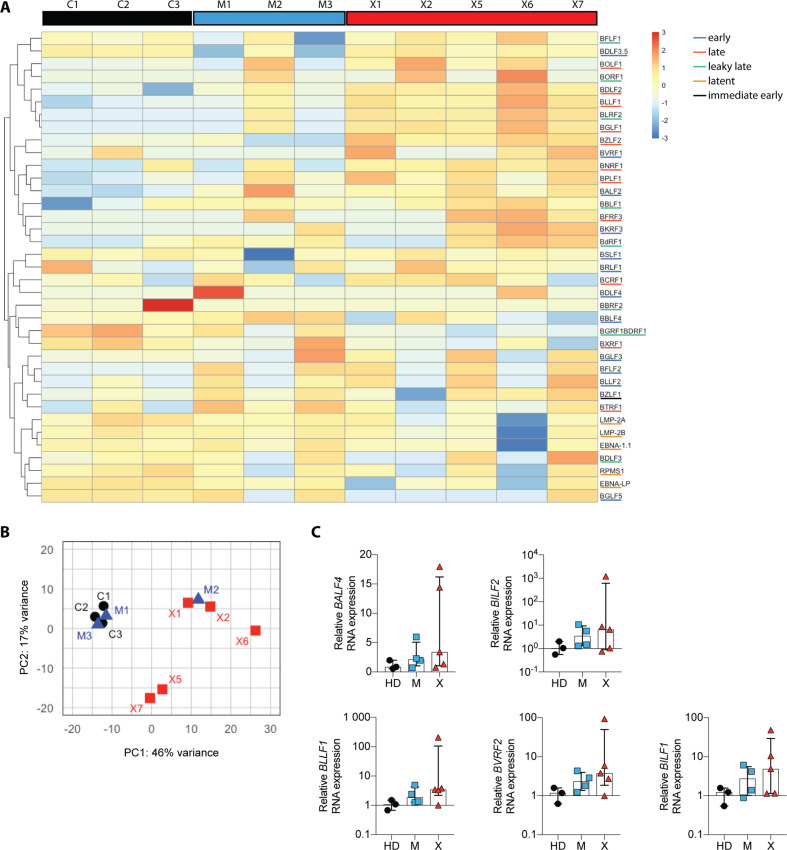
XIAP-deficient LCLs tend to express lytic EBV genes at higher levels. **A** Heatmap of normalized EBV genes in LCLs derived from three healthy donors (C1, C2, and C3), three mothers (M1, M2, and M3) and five XIAP patients (X1, X2, X5, X6, and X7). **B** Principal component analysis of EBV genes. **C** RT-qPCR analysis with previously published primers [79, 80] of late lytic EBV genes in healthy donor (C1, C2, and C3), mother (M1, M2, M3, and M4), and XIAP patient LCLs (X1, X2, X4, X6, and X7). Each dot represents one cell line derived from 1 to 3 technical replicates. Statistical significance was assessed with the Mann–Whitney *U* test.

To investigate the consequences of XIAP loss on cellular and viral gene expression, we performed RNAseq analysis on bulk RNA isolated from LCLs derived from patients, as well as their mothers and wildtype LCLs. In addition to our patients 1 and 2, we included XIAP patient-derived LCLs X5, X6, X7 (Table S2) [26]. Principle component analysis of EBV genes clustered all wild type cells together with M1 and M3, indicating similar expression patterns. Patient-derived LCLs clustered separately, suggesting altered but variable viral gene expression in different XIAP patients. Additionally, the mother of patient 2 reveals a similar gene expression as X2 (Fig. 4B). A heatmap depicting normalized and transformed EBV gene expression suggests that most XIAP patient-derived LCLs express lytic genes at higher levels than mother and healthy donor LCLs (Fig. 4A and Data S2). Genes in the heatmap are categorized and color-coded according to the grouping of Djavadian et al. into latent, immediate early, ‘leaky’ late and late [29]. Genes in the upper half of the heatmap correspond mainly to ‘leaky’ late or late genes, with higher expression in the patient-derived LCLs, such as BDLF2, BLLF1, BLRF2, BGLF1, and BZLF2. To verify the increased expression of lytic genes in XIAP LCLs, we analyzed the expression of some lytic genes, including BALF4, BILF2, BLLF1, BVRF2, and BILF1 by RT-qPCR (Fig. 4C). Although some patient-derived LCLs showed a tendency towards higher expression levels of these genes compared to wild type cells, no statistical significance was reached due to the heterogeneity of this lytic EBV gene expression in LCLs of different patients. Additional analysis of early lytic and latent gene expression also did not show statistically significant differences (Fig. S4A, B). The tendency of higher lytic EBV gene expression did also not result in altered chemokine and cytokine production by XIAP-deficient LCLs (Fig. S4C). Thus, XIAP-deficient LCLs show a tendency towards higher lytic EBV gene expression but this did not significantly affect their growth in vitro or in vivo, as demonstrated above.

### Upregulation of the tumor suppressor CADM1 in XIAP-deficient LCLs

**Fig. 5:**
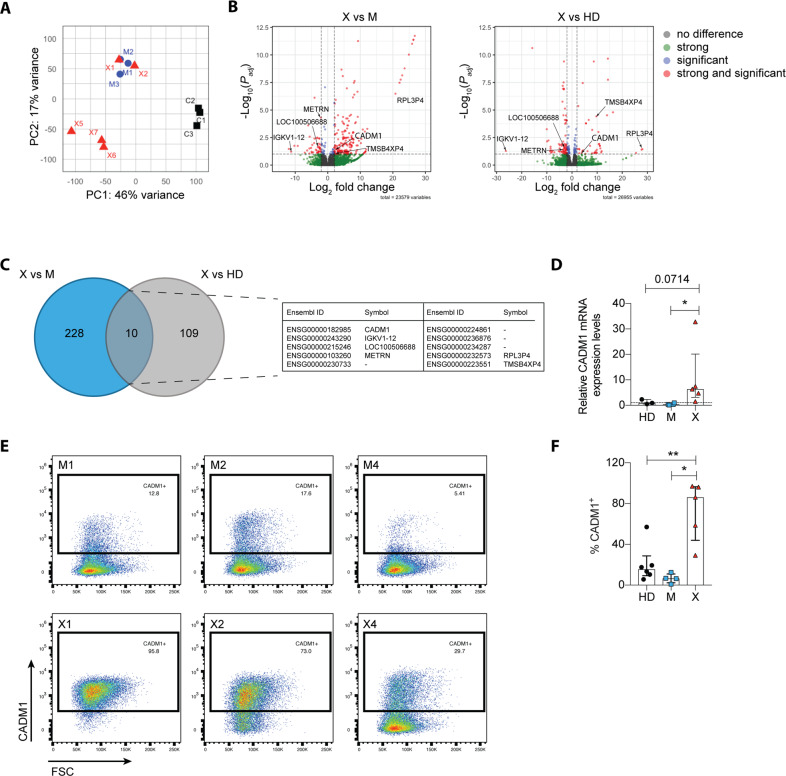
CADM1 expression is upregulated in XIAP patient-derived LCLs compared to healthy donors and mothers. **A** Principal component analysis of cellular gene transcripts in LCLs derived from three healthy donors (C1, C2, and C3), three mothers (M1, M2, and M3), and five XIAP patients (X1, X2, X5, X6, and X7). **B** Volcano plots highlighting differentially expressed genes (DEG) in XIAP patient-derived LCLs relative to mothers (left) and relative to healthy donors (right). The horizontal line corresponds to FDR = 0.1, with genes located above being significantly changed. The two vertical lines correspond to a log_2_ fold change of 2. Genes strongly and significantly expressed are depicted in red. **C** Venn diagram showing overlapping DEG after comparing XIAP patient LCLs to mother or healthy donor LCLs. **D** Relative CADM1 gene expression in healthy donors (*n* = 3), mothers (*n* = 4) and XIAP patients (*n* = 5) as determined by RT-qPCR. **E** Representative flow cytometry plots depicting CADM1 protein expression on the cell surface of three mother-patient pairs. **F** Quantification of CADM1 expression by flow cytometry of all investigated LCLs (HD, *n* = 6; M, *n* = 4; X, *n* = 5). **D**, **F** Each dot represents one donor with the mean expression of three technical replicates (median + IQR). Significance was assessed using the Mann–Whitney *U* test, *p < 0.05, **p < 0.01.

Additionally, we analyzed the host transcriptome of all LCLs and compared patients to mothers and patients to wildtype LCLs. Principal component analysis generated three clusters. One cluster grouped all healthy donor controls together and a second cluster grouped the three mothers together with two patients, which are the respective sons of the mothers. A third cluster grouped the remaining unrelated patients (Fig. 5A). Gene-set enrichment analysis (GSEA) revealed several pathways differentially regulated in XIAP-deficient LCLs, however, no common pathways in both comparisons that would reveal a phenotype in XIAP-deficient LCLs could be determined (Fig. S5D). Differential gene expression analysis comparing mothers and patients revealed 238, hits whereas patients and wild type controls presented 119 hits. The 10 overlapping differentially expressed genes in both comparisons revealed the adhesion molecule and tumor suppressor CADM1 (TSLC-1, NECL2, SynCAM or IgSF4) as significantly upregulated in patient LCLs (Fig. 5B, C; Data S3). In the context of virus-infected cells, CADM1 expression has been shown to be upregulated upon Kaposi sarcoma-associated herpes virus (KSHV) infection of primary B cells and in human T-lymphotropic virus type 1 (HTLV-1)-infected cells [30, 31]. In these virus-infected cells the intracellular domain of CADM1 plays a role as a scaffold to enable chronic signaling through the canonical and non-canonical NF-κB pathway, which is required for cell survival. We hypothesized that CADM1 could play an equal important role in chronic NF-κB activation in LCLs when the XIAP protein is absent. We validated the upregulation of CADM1 mRNA by RT-qPCR (Fig. 5D) and analyzed CADM1 protein expression by flow cytometry (Fig. 5E, F). Indeed, patient-derived LCLs showed higher frequencies of CADM1-positive cells and consistent upregulation of CADM1 on the cell surface in comparison to their respective mothers. Comparison of all 5 XIAP-deficient LCLs to mothers and wild-type controls showed significant higher expression levels of CADM1 in XIAP-deficient LCLs (Fig. 5F). To investigate whether CADM1 expression in patient-derived LCLs is due to the absence of XIAP, we made use of the CRISPR/Cas9 technology to knock-out XIAP in the LCLs derived from the patients’ mothers. We assessed XIAP knock-out efficiency and CADM1 expression by flow cytometry for the first time after five days (Fig. S5A). Knock-out efficiency was between 70 and 80% in each cell line (Fig. S5B). We then investigated whether CADM1 would be upregulated on the XIAP negative cells in comparison to the XIAP positive cells, which act as an internal control. Over the course of 3 weeks post XIAP knock-out, CADM1 expression did however not increase in the XIAP negative population (Fig. S5C). Therefore, XIAP deletion in already established LCLs does not seem to induce the phenotypic changes, like CADM1 upregulation, that are observed upon EBV transformation of XIAP-deficient B cells.

To see whether CADM1 has a pro-survival or pro-proliferative function, similar to the one observed in KSHV-infected cell lines, in established XIAP-deficient LCLs, we again made use of the CRISPR/Cas9 technology to knock-out CADM1. Knock-out efficiency was assessed after 5 days by flow cytometry (Fig. S6A). We then followed their proliferation rate by trypan blue exclusion as well as by cell trace violet (CTV) staining and Ki67 expression (Fig. S6B–E). There was no difference in the growth between CADM1-ko cells and ctrl-ko cells. (Fig. S6B). With the Ki67 and CTV staining we could specifically monitor the CADM1 positive and negative subpopulations (Fig. S6C). Neither Ki67 (Fig. S6D) nor CTV (Fig. S6E) showed a difference between both populations after 5 additional days in culture. In line with these data, CADM1-ko cells were not more prone to apoptosis compared to ctrl-ko cells as determined by AnnexinV/PI staining (Fig. S6E). These results suggest that CADM1 expression in XIAP-deficient LCLs is not required for their survival and proliferation in vitro.

As CADM1 can also mediate heterotypic interactions with T and NK cells through the class I-restricted T cell-associated molecule (CRTAM) and was shown to enhance lysis of HTLV-infected cells [32, 33], we assessed the killing efficiency of XIAP-deficient LCLs by T cells. We generated T cell clones specific for the EBV EBNA1 and EBNA3A antigens and used them in killing assays targeting HLA-matched wild type and XIAP-deficient LCLs. XIAP-deficient LCLs were killed to the same extent as wild type LCLs (Fig. S6G) and cytokine secretion, as well as degranulation markers were expressed at similar levels in the co-cultured T cells in both conditions (Fig. S6H). Thus, XIAP-deficient LCLs did not seem to be more susceptible to cytotoxic T cell killing.

## Discussion

Despite a high susceptibility of XIAP-deficient T cells to cell death after activation, EBV infection causes pathologies only in a third of XLP-2 patients and no EBV-associated lymphoproliferative diseases have so far been reported [2, 3, 27, 34–37]. This is surprising because compromised cytotoxic CD8^+^ T cell function has been identified in many other primary immunodeficiencies as the cause of immune control loss against EBV-associated lymphomagenesis [38–41]. XLP-1 patients are exemplary for this phenomenon and 20–30% of these patients go on to develop EBV-associated lymphomas [42]. The importance of SAP-mediated 2B4 signaling in T cells was already demonstrated by our lab using the humanized mouse model of EBV infection [43]. In this model, 2B4 receptor blockade resulted in increased tumorigenesis, demonstrating the utility of this model in studying primary immunodeficiency diseases. Shedding some light on this discrepancy in XLP-2 patients, we report here that B cell frequency is reduced upon IAP inhibition and the memory B cell compartment appears compromised in XLP-2 patients. Since EBV exploits this B cell differentiation for persistence [44], this might in part explain the absence of EBV-associated malignancies in XLP-2 patients that originate from germinal center B cells, such as Hodgkin’s and Burkitt’s lymphoma [45]. The fact that the frequency of mice harboring tumors did not increase after IAP depletion confirms that XIAP loss does not promote EBV-associated lymphomagenesis. Furthermore, we demonstrate the upregulation of the tumor suppressor CADM1 upon EBV-mediated transformation of B cells from XLP-2 patients, resulting in LCLs that resemble extrafollicular lymphoproliferative diseases caused by EBV [46].

Even though our current experimental systems were not able to pinpoint how CADM1 expression might compromise EBV-associated lymphomagenesis in the absence of XIAP, it might stimulate immune control, possibly by innate immunity in secondary lymphoid tissues to prevent EBV-positive lymphomas in XLP-2 patients.

XIAP has a critical role in survival of myeloid cells. In the absence of XIAP, myeloid cells are particularly prone to cell death in response to infection [47–49]. Stimulation with TNF super family receptors, such as Fas or TNFR2, myeloid cells die either by necroptosis or via inflammasome activation [50, 51], and any stimuli which also decreases cIAP1/2 protein levels, sensitize *xiap*^−/−^ macrophages to TNF-mediated cell death [52]. In a XIAP-deficient patient, macrophages may provide an inflammatory environment, eliciting an immune response and thereby preventing tumor formation.

CADM1 has been found upregulated on lymphocytes that are transformed with two other human tumorviruses, namely HTLV-1 and KSHV or human herpesvirus 8 (HHV8) [30–32, 53, 54]. Both cell intrinsic and extrinsic effects of CADM1 in the respective T and B cell lymphomas have been described. For HTLV-1-associated T cell lymphomas and KSHV-associated primary effusion lymphoma, CADM1 has been described to support viral oncogene-driven NF-κB activation, and thereby to directly drive transformed lymphocyte proliferation [30, 31]. In addition, CADM1 mediates cytotoxic lymphocyte recognition of tumor cells, including HTLV-1-associated CD4^+^ T cell lymphomas [32, 33]. We tested those two mechanisms for XIAP-deficient EBV-transformed B cells, but deletion of CADM1 in LCLs of XLP-2 patients did not significantly change their growth, nor did cytotoxic CD8^+^ T cell recognition significantly differ between XIAP-deficient and sufficient LCLs. Thus, CADM1 might mediate its tumor suppressor function against EBV-transformed B cells during XIAP deficiency in a cell extrinsic function that is independent of CD8^+^ T cells.

The tumor suppressor function of CADM1, however, extends beyond virus-induced lymphomas. Along these lines, CADM1-deficient mice develop lymphomas and to a lesser degree leukemias, as well as epithelial cell-derived adenomas spontaneously at increased incidences [55]. Accordingly, CADM1 is frequently silenced in acute lymphoblastic leukemias [56]. Furthermore, CADM1 is deleted in a subset of myelodysplastic syndromes [57]. Beyond hematopoietic malignancies, it was originally described as tumor suppressor in lung cancer 1 (TSLC-1) in non-small-cell lung cancer [58]. However, its inactivation is also associated with prostate, esophageal, gastric, nasopharyngeal, breast, and cervical carcinoma, as well as meningioma [59–67]. Especially, invasive tumors upregulate CADM1 [68–71]. Thus, CADM1 downregulation is observed in a variety of hematopoietic and epithelial malignancies, for which also loss of cell adhesion and of immune surveillance are discussed as possible mechanisms. Therefore, the CADM1 upregulation observed in this study on EBV-transformed B cells of XLP-2 patients might be in part responsible for the absence of EBV-associated lymphoproliferative diseases despite compromised EBV-specific immune control in the absence of XIAP.

## Materials and methods

### Humanized mice generation, infection, and SMAC-mimetic treatment

NOD-*scid* γ_c_^−/−^ (NSG) mice (Jackson Laboratory, Bar Harbor, ME, USA) were bred and maintained under specific pathogen-free conditions at the Institute of Experimental Immunology, University of Zurich, Switzerland. Newborn pups, 1 to 5 days of age, were sub-lethally irradiated with 1 Gy by X-ray. Five to seven hours later, the pups were injected intrahepatically with 1–3 × 10^5^ CD34^+^ hematopoietic stem and progenitor cells (HPSCs) derived from human fetal livers (Advanced Bioscience Resources). CD34^+^ HPSCs were isolated by positive selection of CD34^+^ cells by magnetic cell sorting (Miltenyi), according to the manufacturer’s protocol and as described previously [21]. Three months later, the engraftment of human immune cells was assessed in the peripheral blood by flow cytometry. Male and female mice were intraperitoneally (i.p.) infected with 10^5^ Raji Infectious Units (RIU) of the EBV B95-8 strain or PBS control and followed for up to 5 weeks post-infection. For treatment with SMAC-mimetics, mice were evenly distributed to different treatment groups according to their human reconstitution levels in the blood and with an equal male to female ratio. The SMAC-mimetics Birinapant (Chemietek, Indianapolis, IN, USA) and GT13072 (Tetralogics, Malvern, PA, USA) were dissolved in 12.5% Captisol (Captisol, KS, USA). Aliquots of the compounds were stored at −80 °C and freshly thawed on each day of injection. Mice were treated intraperitoneally three times a week during the whole experiment at 50 mg/kg/dose, starting with two injections prior to EBV infection. Control mice were treated with equal amounts of solvent (12.5% Captisol). Investigators were not blinded regarding the different treatment groups of the animal experiments. Organs of the animals were harvested after 4 to 5 weeks post infection or earlier if the mice developed general health symptoms and/or weight loss that required their pre-mature euthanasia according to the general guidelines of the animal welfare protocol. Mice that had to be euthanized prematurely, before 3 weeks of infection as well as mice that did not get infected as assessed by viral loads in the blood and spleen, were excluded from the analysis. Tumor development was assessed at the day of sacrifice by macroscopic inspection of the organs and peritoneum.

### Production of recombinant EBV

The EBV strain B95-8 was produced in HEK293 cells (ATCC) containing a GFP-encoding wild type EBV BACmid (p2089; kind gift from W. Hammerschmidt) (Delecluse et al., 1998). Recombinant virus particles were collected from the supernatant three days after lytic cycle induction by transfecting the cells with the BZLF1 (p509) and BALF4 (p2670) -encoding plasmids with METAFECTENE® PRO (Biontex). Supernatants were collected and filtered through a 0.45 μm filter and then concentrated at 30 000xG, for 2 h at 4 °C. Virus concentrates were titrated on Raji cells (ATCC) as previously described [72]. Raji Infectious Units (RIU) were determined by measuring GFP-positive cells two days after infection by flow cytometry on a BD FACSCanto II.

### Viral load quantification in blood and tissue

DNA from whole blood and serum was extracted with the NucliSENS EasyMAG System (Biomérieux) and DNA from splenic tissues was isolated using the DNeasy Blood and Tissue kit (QIAGEN), according to the manufacturer’s recommendations. Taqman real-time PCR was used to quantify EBV viral loads using modified primers (5′-CTTCTCAGTCCAGCGCGTTT-3′ and 5′-CAGTGGTCCCCCTCCCTAGA-3′) and a fluorogenic probe (5′-FAM CGTAAGCCAGACAGCAGCCAATTGTCAG-TAMRA-3′) to detect the conserved EBV BamHI-W fragment. Blood and splenic tissue viral loads from SMAC-mimetic treated mice were analyzed on an ABI Prism 7700 Sequence detector (Applied BioSystems) and run in duplicates, with EBV loads indicated in EBV genome copy numbers. EBV load in serum of SMAC-mimetic treated mice and LCL-transfer experiment samples were analyzed in triplicates and run on a CFX384 Touch Real-time PCR Detection System (Bio-Rad), indicating EBV International Units (IU).

### Flow cytometric analysis of huNSG cells

Peripheral blood from mice was collected either by tail vein bleeding or terminal heart puncture at the day of sacrifice. An aliquot of whole blood was used to determine the whole blood cell count with a Beckman Coulter AcT diff Analyzer for the calculation of total cell counts. Red blood cells were lysed with ACK-lysis buffer for 5 min and subsequently stained with fluorochrome-conjugated antibodies for phenotypic analysis by flow cytometry. Cells were stained with surface markers for 20 min at 4 °C. Human reconstitution levels were determined with the expression of human CD45, CD3, CD4, CD8, CD19, and NKp46. Labeled cells were acquired on a BD FACSCantoII, BD LSRFortessa, or BD FACSymphony. Data analysis was performed using FlowJo software (FlowJo LLC).

### Flow cytometric analysis of cell lines

Lymphoblastoid cell lines (LCLs) or other cell lines were washed in 1× PBS before the addition of Fc block (BD) and a fixable live/dead stain (BioLegend) and incubated for 10 min at room temperature. CADM1 expression was determined by staining cells with α-CADM1 or normal chicken isotype-control (MBL International Corporation), followed by goat α-chicken-AF647 secondary antibody (Abcam). Positive gates were set according to isotype-control staining and unspecific staining was subtracted from the CADM1 signal for quantification. XIAP protein expression was analyzed by staining with α-XIAP (clone 48, BD) for 20 min at 4 °C after fixation and permeabilization with the Cytofix/Cytoperm intracellular staining kit (BD) for 15 min at 4 °C. A secondary α-mouse IgG-PE (A85-1, BD Pharmingen) antibody was used and incubated for 20 min at 4 °C. Stained cells were acquired either on a BD LSRFortessa or BD FACSymphony. The data was analyzed using FlowJo software (FlowJo LLC) All antibodies and dyes used for flow cytometry are listed in Table S5.

### Processing of whole blood samples

Venous blood was drawn into EDTA tubes and processed on the same day. A small aliquot of the whole blood was used to determine the white blood cell count with a Beckman Coulter AcT diff Analyzer. Additional whole blood aliquots were used for immune cell phenotyping after red blood cell lysis with ACK-lysis buffer for 5 min and staining with antibodies for 20 min at 4 °C. Labeled cells were acquired either on a BD LSRFortessa or BD FACSymphony and data analysis was performed using FlowJo software (FlowJo LLC). Peripheral blood mononuclear cells (PBMCs) were isolated from the remaining blood samples by Ficoll-Paque (GE Healthcare), according to the manufacturer’s protocol.

### Lymphoblastoid cell line generation

For the generation of LCLs, B cells were isolated from PBMCs by positive selection using the MACS technology with CD19 microbeads (Miltenyi), according to the manufacturer’s protocol. Isolated B cells were resuspended in R10 (RPMI 1640 + 10% FBS, 1% penicillin/streptomycin + 1% L-Glutamine Gibco, Thermo Fisher Scientific). EBV particles were added at a MOI of 0.1 to the cells, which were then incubated at 37 °C, 5% CO_2_ until EBV-transformed B cells grew out. Established LCLs were passaged 2–3 times per week. LCLs provided by R. Marsh were generated in a similar way, by infecting total PBMCs with EBV collected from the spent supernatant of the B95-8 marmoset cell line and the addition of cyclosporine A. All cell lines were routinely checked for mycoplasma contamination.

### T cell clone generation

EBV-specific T cell clones for EBNA1 and EBNA3A were generated from PBMCs derived from a healthy donor or from mother 2. PBMCs were stimulated with peptide-pulsed (1 μM; EBNA1_407–__417_: HPVGEADYFEY and HLA-B35 restricted; EBNA3A_325–__333_: FLRGRAYGL and HLA-B8 restricted) autologous LCLs for 3–4 h and IFN-γ secreting cells were then isolated with the IFN-γ Secretion Assay – Detection kit (Miltenyi Biotech). Isolated cells were then seeded into 96-well plates using the single cell cloning dilution method. PBMCs, stimulated overnight with PHA (5 μg/ml), and peptide-pulsed LCLs were irradiated at 20 and 60 Gy, respectively, and served as feeder cells. IL-2 (Peprotech) was added after 2 days and subsequently two times a week at 125 U/ml. After 3–4 weeks, colonies formed, which were screened for IFN-γ production by ELISA, after stimulation with 1 μM specific peptide. Positive clones were kept in culture and re-fed every 2–3 weeks as described above.

### In vitro T cell killing and cytokine secretion assays

EBNA1 and EBNA3A-specific T cell clones were co-cultured with autologous wild type or XIAP-deficient LCLs to assess the killing efficiency of the LCLs. The target LCLs were labeled with PKH-26 (Sigma-Aldrich) for 5 min according to the manufacturer’s protocol to distinguish them from the T cells. LCLs were then co-cultured with the T cell clones at a 5:1 effector:target ratio and incubated for 18 h. LCL cell death was assessed by adding TO-PRO-3-iodide (ThermoFisher Scientific) at a final concentration of 0.5 μM prior to acquisition.

To determine the degranulation capacity by CD107a (H4A3, BD Biosciences) and the ability of cytokine secretion, T cell clones were co-cultured as described above for 6 h. After 2 h Brefeldin A (Sigma-Aldrich) was added and cells were cultured for additional 4 h. Cytokine production was measured by intracellular staining of TNF-α (Mab11, BD Biosciences), IL-2 (MQ1-17H12, eBioscience) and IFN-γ (4S.B3, eBioscience).

### NOD2 stimulation of monocytes

XIAP-blockade by GT13072 was shown by preincubating buffy-coat derived monocytes with Birinapant or GT13072 for 30 min, before stimulating the monocytes with the NOD2 ligand L18-MDP (InvivoGen) as previously described [23]. Briefly, PBMCs from buffy coats were seeded and cultured overnight in a 24-well plate. Non-adherent cells were washed away and adherent cells were treated with GT13072 or Birinapant with indicated concentrations for 30 min. L18-MDP was then added to each condition at 200 ng/mL, together with BrefeldinA for 2.5 h. TNF-α secretion was assessed by intracellular cytokine staining and acquired on a BD LSRFortessa.

### Cytokine and chemokine measurement

IFN-γ secretion of T cell clones was measured in the supernatant using the IFN-γ ELISA kit (3420-1H-20, MABTECH) following the manufacturer’s protocol. Total IgM from mouse-serum was detected using the IgM-ELISA kit (3880-1AD-6, MABTECH) according to the manufacturer’s protocol. Serum samples were pre-diluted 1:10 000. IL-27 in cell culture supernatant was analyzed using the IL-27 (3458-1H-6, MABTECH) kit, following the manufacturers protocol. CXCl9, CXCL10, IL-6, and IL-10 were analyzed with a Magnetic Luminex assay kit (Bio-Techne, Minneapolis, MN, USA). The measurements were performed on a Bio-Rad BioPlex 200 system (Bio-Rad Laboratories, Basel, Switzerland) and data were analyzed with the Bio-Plex software 6.0 (version 6.0, Bio-Rad).

### LCL cell lines used for RNAseq analysis

All LCLs and data used for the RNAseq analysis are listed in Table S2. LCLs from patient 1 and 2, as well as LCLs from mothers 1, 2, and 3 were generated for this study as described above. XIAP patient LCLs number 5, 6, and 7 were kind gifts of Rebecca Marsh (Cincinnati Children’s Hospital, USA). The previously generated RNA-read count data set of three healthy male donors [22] was used as healthy controls.

### High-throughput sequencing and RNAseq analysis

LCL RNA was isolated as described below with the RNeasy Mini kit (QIAGEN), with on-column DNase digestion (RNase-Free DNase Set, QIAGEN). After mRNA-isolation with the NEBNext Poly(A) mRNA Magnetic Isolation Module, strand-specific RNA-Seq libraries were generated using the NEXTflex rapid Directional qRNA-Seq Kit (Bioo Scientific). Sequencing of the libraries was performed on an Illumina NextSeq 500 platform (single read 75) at an average of ~20 million reads. Three replicates of each donor were sequenced and read counts were combined, resulting in ~60 million reads per donor, for further analysis (see Data S1 for sequencing depth).

For differential gene expression analysis, gene annotations from gencode (version 33) for GRCh38 assembly were used and for the EBV GenBank annotations for reference sequence NC_007605 (NCBI: NC_007605.1) were used. The data was imported with the R package tximport [73]. Gene abundance was quantified using Salmon (v1.5.1) [74]. Normalization of read counts and differential expression (DE) analysis was performed using the DESeq2 package [75]. P-values for differentially expressed genes were calculated by re-estimating the null variance of the Wald test statistic output from DESeq2 with the R package fdrtool [76]. The Benjamini–Hochburg method was used to adjust the p-value (FDR). FDR (BH-adjusted *p*-values) < 0.1 and Log_2_ fold change of 2 was used as criteria for the final DE gene list. Heatmaps were generated using variance stabilizing transformation [77] on normalized counts of genes, and R package pheatmap was used with Pearson correlation as a distance measure for rows clustering. Volcano plots were generated using the R package EnhancedVolcano. R statistical computing language (version 4.1.1) was used to perform above-mentioned steps, except gene quantification. Gene Set Enrichment Analysis (GSEA) was performed using the online tool WebGestalt.

### Quantitative RT-PCR (qRT-PCR)

RNA was isolated from cultured cells with the RNeasy Mini kit (QIAGEN), following the manufacturer’s protocol. To prevent contamination from genomic DNA, on-column DNase digest was performed during RNA isolation (RNase-Free DNase Set, QIAGEN). Complementary DNA (cDNA) was generated using oligo(dT)s for cellular genes or specific primer mixes for EBV genes at a final concentration of 10 μM in a 20 μl reaction volume with the GoScript Reverse Transcriptase (Promega). Cellular gene expression was analyzed with SYBR Select Master Mix for CFX (Thermofisher) and run on a CFX384 Touch Real-Time PCR Detection System (Bio-Rad) using a program of 2 min at 50 °C and 2 min at 95 °C, followed by 40 cycles of amplification (95 and 54 °C for 15 s each) with a final extension at 72 °C for 1 min. Gene expression of CADM1 was calculated relative to the geometric mean of the housekeeping gene *GAPDH* and presented as a relative expression. EBV gene expression was analyzed with TaqMan Universal PCR Master Mix (Applied Biosystems) using a protocol of 2 min at 50 °C and 10 min at 95 °C, followed by 50 cycles of amplification (95 °C for 15 s and 60 °C for 1 min) [22] Relative gene expression was calculated to the geometric mean of the reference gene *SDHA* (TaqMan Applied Biosystems Gene Expression Assay (Hs00417200). Primers used for qRT-PCR are listed in Table S3.

### Gene editing with CRISPR/Cas9

Gene knockouts of CADM1 and XIAP were generated using the 4D-Nucleofector system (Lonza) with the P3 primary cell kit (Lonza). Guide RNAs were generated by mixing Alt-R® CRISPR-Cas9 tracrRNA and predesigned Alt-R® CRISPR-Cas9 crRNA (IDT) at equimolar concentrations to a final duplex concentration of 100 μM and incubating them at 95 °C for 5 min. crRNAs used in this study are listed in Table S4. RNP complexes were subsequently generated by mixing gRNAs with Alt-R® S.p. Cas9 Nuclease V3 in a 5:1 molar ratio. 0.5 × 10^6^ cells were harvested and washed in 1× PBS and resuspended in the P3 solution. The previously generated RNP was added, resulting in a 20 μl reaction volume. Cells were immediately electroporated using the DN-100 program and allowed to rest for 10 min after addition of 80 μl R10. Edited cells were then seeded into a 96-well plate and incubated at 37 °C, 5% CO_2_ for 5 days. Knockout efficiency was determined by analysis of protein expression by flow cytometry as described above.

### Cell death and proliferation analysis

Treated cells were collected and washed once in PBS. Cells were then stained with Annexin V and PI in 1× Annexin V staining buffer (eBioscience) for 15 min at room temperature in the dark. Samples were acquired within 1 hour after staining. For proliferation assays, LCLs were stained with cell trace violet (CTV) according to the manufacturer’s recommendations and seeded at a density of either 0.3 or 0.5 million cells per well and incubated for up to 4 days at 37 °C, 5% CO_2_. On the day of analysis, cells were counted by trypan blue exclusion and stained with NIR or Aqua live/dead stain (BioLegend). For additional analysis of Ki67 expression in cultured LCLs, cells were fixed and permeabilized for 20 min at 4 °C and stained with a Ki67 antibody for 1 h at room temperature, using the FoxP3/Transcription Factor Staining Buffer Set (eBioscience). Processed samples were acquired on a BD FACSCantoII or BD LSRFortessa. The data was analyzed using FlowJo software (FlowJo LLC).

### Western blot

Organs from mice (lungs, spleens) were lysed on ice for 30 min in DISC lysis buffer (20 mM Tris-HCl pH 7.5, 150 mM NaCl, 10% (v/v) glycerol, 1% (v/v) NP-40, 2 mM EDTA, 5 mM EGTA, 30 mM NaF, 40 mM b-glycerophosphate pH 7.2, 10 mM sodium pyrophosphate, 2 mM activated sodium orthovanadate, protease inhibitors) as previously described [78]. Cell lines were lysed in RIPA buffer (Sigma-Aldrich) supplemented with protease inhibitors (cOmplete^™^, EDTA-free Protease Inhibitor Cocktail, Roche). The lysate was pelleted by centrifuging for 20 min at 4 °C. The supernatant was mixed with 4X Lämmli buffer and boiled for 5 min at 95 °C. The protein concentration was determined by Pierce™ BCA Protein Assay Kit (Thermofischer) following the manufacturer’s protocol. 10–30 μg of protein were loaded on 8% SDS-gels and run using TGS buffer. Proteins were transferred to PVDF blotting membranes (Amersham Hybond P 0.45, GE Healthcare) and blocked for 1 h at room temperature in 1% PBS-Tween-20 (PBS-T) with 5% milk powder. Primary antibodies were diluted in PBS-T with 5% milk powder and incubated overnight at 4 °C. Membranes were washed three times for 10 min in PBS-T, followed by incubation with the respective HRP-coupled secondary antibody diluted in PBS and incubated for 1 h at room temperature. After additional three rounds of washing in PBS-T protein bands were detected by chemiluminescence (WesternBright Sirius ECL, Witec AG) on a Fusion FX7 (Witec AG, Heitersheim, Germany). All antibodies used for Western blotting are listed in Table S5.

### Statistical analysis

GraphPad Prism Software was used for statistical analysis. The Mann–Whitney *U* test was used to analyze unpaired data with a non-Gaussian distribution. Error bars indicate median with interquartile range, unless otherwise stated. Normality of data was tested with D’Agostino–Pearson omnibus K2 normality test or Shapiro–Wilk test. Frequencies in more than one category were analyzed by contingency tables and Fisher’s exact test. A *p* value of < 0.05 was considered statistically significant.

## Supplementary information

supplemental figure legends

supplemental Figure 1

supplemental Figure S2

supplemental Figure S3

supplemental Figure S4

supplemental Figure S5

supplemental Figure S6

supplemental Table S1

supplemental Table 2

supplemental Table 3

supplemental Table 4

supplemental Table 5

supplemental Data S1

supplemental Data S2

supplemental Data S3

Original Data File

Nature press checklist

## Data Availability

RNA-seq data can be viewed in Data S1, S2, and S3 and have been deposited in the European Nucleotide Archive (ENA) at EMBL-EBI under accession number PRJEB56290.

## References

[1] Mudde ACA, Booth C, Marsh RA. Evolution of our understanding of XIAP deficiency. Front Pediatr. 2021. https://www.frontiersin.org/articles/10.3389/fped.2021.660520/full. 10.3389/fped.2021.660520 PMC8247594 34222142

[2] Rigaud S, Fondanèche MC, Lambert N, Pasquier B, Mateo V, Soulas P (2006). XIAP deficiency in humans causes an X-linked lymphoproliferative syndrome. Nature.

[3] Schmid JP, Canioni D, Moshous D, Touzot F, Mahlaoui N, Hauck F (2011). Clinical similarities and differences of patients with X-linked lymphoproliferative syndrome type 1 (XLP-1/SAP deficiency) versus type 2 (XLP-2/XIAP deficiency). Blood.

[4] Silke J, Vucic D. IAP family of cell death and signaling regulators. Methods in Enzymology 2014;545:35–65. 10.1016/B978-0-12-801430-1.00002-0. 10.1016/B978-0-12-801430-1.00002-0 25065885

[5] Duckett CS, Nava VE, Gedrich RW, Clem RJ, Van Dongen JL, Gilfillan MC (1996). A conserved family of cellular genes related to the baculovirus iap gene and encoding apoptosis inhibitors. EMBO J.

[6] Eckelman BP, Drag M, Snipas SJ, Salvesen GS (2008). The mechanism of peptide-binding specificity of IAP BIR domains. Cell Death Differ.

[7] Shiozaki EN, Chai J, Rigotti DJ, Riedl SJ, Li P, Srinivasula SM (2003). Mechanism of XIAP-mediated inhibition of Caspase-9. Mol Cell.

[8] Lu M, Lin SC, Huang Y, Kang YJ, Rich R, Lo YC (2007). XIAP induces NF-κB activation via the BIR1/TAB1 interaction and BIR1 dimerization. Mol Cell.

[9] Gyrd-Hansen M, Darding M, Miasari M, Santoro MM, Zender L, Xue W (2008). IAPs contain an evolutionarily conserved ubiquitin-binding domain that regulates NF-κB as well as cell survival and oncogenesis. Nat Cell Biol.

[10] Schwerd T, Pandey S, Yang HT, Bagola K, Jameson E, Jung J (2017). Impaired antibacterial autophagy links granulomatous intestinal inflammation in Niemann–Pick disease type C1 and XIAP deficiency with NOD2 variants in Crohn’s disease. Gut.

[11] Gradzka S, Thomas OS, Kretz O, Haimovici A, Vasilikos L, Wong WWL, et al. Inhibitor of apoptosis proteins are required for effective fusion of autophagosomes with lysosomes article. Cell Death Dis. 2018. 10.1038/s41419-018-0508-y. 10.1038/s41419-018-0508-y PMC5943300 29743550

[12] Huang X, Wu Z, Mei Y, Wu M (2013). XIAP inhibits autophagy via XIAP-Mdm2-p53 signalling. EMBO J.

[13] Oberoi TK, Dogan T, Hocking JC, Scholz RP, Mooz J, Anderson CL (2012). IAPs regulate the plasticity of cell migration by directly targeting Rac1 for degradation. EMBO J.

[14] Liu J, Zhang D, Luo W, Yu J, Li J, Yu Y (2012). E3 ligase activity of XIAP RING domain is required for XIAP-mediated cancer cell migration, but not for its RhoGDI binding activity. PLoS One.

[15] Chang YC, Cheung CHA (2021). An updated review of smac mimetics, lcl161, birinapant, and gdc-0152 in cancer treatment. Appl Sci.

[16] Du C, Fang M, Li Y, Li L, Wang X (2000). Smac, a mitochondrial protein that promotes cytochrome c-dependent caspase activation by eliminating IAP inhibition. Cell.

[17] Verhagen AM, Ekert PG, Pakusch M, Silke J, Connolly LM, Reid GE (2000). Identification of DIABLO, a mammalian protein that promotes apoptosis by binding to and antagonizing IAP proteins. Cell.

[18] Obexer P, Ausserlechner MJ (2014). X-linked inhibitor of apoptosis protein—a critical death resistance regulator and therapeutic target for personalized cancer therapy. Front Oncol.

[19] Condon SM, Mitsuuchi Y, Deng Y, Laporte MG, Rippin SR, Haimowitz T (2014). Birinapant, a smac-mimetic with improved tolerability for the treatment of solid tumors and hematological malignancies. J Med Chem.

[20] Fan LX, Zhou X, Sweeney WE, Wallace DP, Avner ED, Grantham JJ (2013). Smac-mimetic-induced epithelial cell death reduces the growth of renal cysts. J Am Soc Nephrol.

[21] Strowig T, Gurer C, Ploss A, Liu YF, Arrey F, Sashihara J (2009). Priming of protective T cell responses against virus-induced tumors in mice with human immune system components. J Exp Med.

[22] McHugh D, Caduff N, Barros MHM, Rämer PC, Raykova A, Murer A (2017). Persistent KSHV infection increases EBV-associated tumor formation in vivo via enhanced EBV lytic gene expression. Cell Host Microbe.

[23] Ammann S, Elling R, Gyrd-Hansen M, Dückers G, Bredius R, Burns SO (2014). A new functional assay for the diagnosis of X-linked inhibitor of apoptosis (XIAP) deficiency. Clin Exp Immunol.

[24] Noonan AM, Bunch KP, Chen J-Q, Herrmann MA, Lee J-M, Kohn EC (2016). Pharmacodynamic markers and clinical results from the phase 2 study of the SMAC mimetic birinapant in women with relapsed platinum-resistant or -refractory epithelial ovarian cancer. Cancer.

[25] Seung E, Tager AM (2013). Humoral immunity in humanized mice: A work in progress. J Infect Dis..

[26] Marsh RA, Madden L, Kitchen BJ, Mody R, McClimon B, Jordan MB (2010). XIAP deficiency: A unique primary immunodeficiency best classified as X-linked familial hemophagocytic lymphohistiocytosis and not as X-linked lymphoproliferative disease. Blood.

[27] Speckmann C, Lehmberg K, Albert MH, Damgaard RB, Fritsch M, Gyrd-Hansen M (2013). X-linked inhibitor of apoptosis (XIAP) deficiency: The spectrum of presenting manifestations beyond hemophagocytic lymphohistiocytosis. Clin Immunol.

[28] Mrozek-Gorska P, Buschle A, Pich D, Schwarzmayr T, Fechtner R, Scialdone A (2019). Epstein–Barr virus reprograms human B lymphocytes immediately in the prelatent phase of infection. Proc Natl Acad Sci USA.

[29] Djavadian R, Hayes M, Johannsen E (2018). CAGE-seq analysis of Epstein-Barr virus lytic gene transcription: 3 kinetic classes from 2 mechanisms. PLoS Pathog.

[30] Pujari R, Hunte R, Thomas R, van der Weyden L, Rauch D, Ratner L (2015). Human T-cell leukemia virus type 1 (HTLV-1) tax requires CADM1/TSLC1 for inactivation of the NF-κB inhibitor A20 and constitutive NF-κB signaling. PLoS Pathog.

[31] Hunte R, Alonso P, Thomas R, Bazile CA, Ramos JC, van der Weyden L (2018). CADM1 is essential for KSHV-encoded vGPCR-and vFLIP-mediated chronic NF-κB activation. PLoS Pathog.

[32] Manivannan K, Rowan AG, Tanaka Y, Taylor GP, Bangham CRM (2016). CADM1/TSLC1 identifies HTLV-1-infected cells and determines their susceptibility to CTL-mediated lysis. PLoS Pathog.

[33] Boles KS, Barchet W, Diacovo T, Cella M, Colonna M (2005). The tumor suppressor TSLC1/NECL-2 triggers NK-cell and CD8+ T-cell responses through the cell-surface receptor CRTAM. Blood.

[34] Yang X, Kanegane H, Nishida N, Imamura T, Hamamoto K, Miyashita R (2012). Clinical and genetic characteristics of XIAP deficiency in Japan. J Clin Immunol.

[35] Damgaard RB, Nachbur U, Yabal M, Wong WWL, Fiil BK, Kastirr M (2012). The Ubiquitin Ligase XIAP Recruits LUBAC for NOD2 Signaling in Inflammation and Innate Immunity. Mol Cell.

[36] Damgaard RB, Fiil BK, Speckmann C, Yabal M, Stadt Uzur, Bekker-Jensen S (2013). Disease-causing mutations in the XIAP BIR2 domain impair NOD2-dependent immune signalling. EMBO Mol Med.

[37] Sbihi Z, Tanita K, Bachelet C, Bole C, Jabot-Hanin F, Tores F, et al. Identification of germline non-coding deletions in XIAP gene causing XIAP deficiency reveals a key promoter sequence. J Clin Immunol. 2022. 10.1007/s10875-021-01188-z. 10.1007/s10875-021-01188-z 35000057

[38] Damania B, Münz C (2019). Immunodeficiencies that predispose to pathologies by human oncogenic γ-herpesviruses. FEMS Microbiol Rev.

[39] Fournier B, Latour S (2021). Immunity to EBV as revealed by immunedeficiencies. Curr Opin Immunol.

[40] Latour S, Fischer A (2019). Signaling pathways involved in the T-cell-mediated immunity against Epstein-Barr virus: Lessons from genetic diseases. Immunol Rev.

[41] Tangye SG, Latour S (2020). Primary immunodeficiencies reveal the molecular requirements for effective host defense against EBV infection. Blood.

[42] Nichols KE, Ma CS, Cannons JL, Schwartzberg PL, Tangye SG (2005). Molecular and cellular pathogenesis of X-linked lymphoproliferative disease. Immunol Rev.

[43] Chijioke O, Marcenaro E, Moretta A, Capaul R, Münz C (2015). Role of the 2B4 receptor in CD8+ T-cell-dependent immune control of Epstein-Barr virus infection in mice with reconstituted human immune system components. J Infect Dis.

[44] Münz C (2019). Latency and lytic replication in Epstein–Barr virus-associated oncogenesis. Nat Rev Microbiol.

[45] Shannon-Lowe C, Rickinson A (2019). The global landscape of EBV-associated tumors. Front Oncol.

[46] Farrell PJ (2019). Epstein–Barr virus and cancer. Annu Rev Pathol Mech Dis.

[47] Bauler LD, Duckett CS, O’Riordan MXD (2008). XIAP regulates cytosol-specific innate immunity to Listeria infection. PLoS Pathog.

[48] Prakash H, Albrecht M, Becker D, Kuhlmann T, Rudel T (2010). Deficiency of XIAP leads to sensitization for Chlamydophila pneumoniae pulmonary infection and dysregulation of innate immune response in mice. J Biol Chem.

[49] Hsieh W-C, Chuang Y-T, Chiang I-H, Hsu S-C, Miaw S-C, Lai M-Z (2014). Inability to resolve specific infection generates innate immunodeficiency syndrome in Xiap-/- mice. Blood.

[50] Wicki S, Gurzeler U, Wei-Lynn Wong W, Jost PJ, Bachmann D, Kaufmann T (2016). Loss of XIAP facilitates switch to TNFα-induced necroptosis in mouse neutrophils. Cell Death Dis.

[51] Knop J, Spilgies LM, Rufli S, Reinhart R, Vasilikos L, Yabal M (2019). TNFR2 induced priming of the inflammasome leads to a RIPK1-dependent cell death in the absence of XIAP. Cell Death Dis.

[52] Wong WW-L, Vince JE, Lalaoui N, Lawlor KE, Chau D, Bankovacki A (2014). cIAPs and XIAP regulate myelopoiesis through cytokine production in an RIPK1- and RIPK3-dependent manner. Blood.

[53] Sasaki H, Nishikata I, Shiraga T, Akamatsu E, Fukami T, Hidaka T (2005). Overexpression of a cell adhesion molecule, TSLC1, as a possible molecular marker for acute-type adult T-cell leukemia. Blood.

[54] Takenouchi N, Tanaka M, Sato T, Yao J, Fujisawa JI, Izumo S (2020). Expression of TSLC1 in patients with HAM/TSP. J Neurovirol.

[55] van der Weyden L, Arends MJ, Rust AG, Poulogiannis G, McIntyre RE, Adams DJ (2012). Increased tumorigenesis associated with loss of the tumor suppressor gene Cadm1. Mol Cancer.

[56] Paulsson K, An Q, Moorman AV, Parker H, Molloy G, Davies T (2009). Methylation of tumour suppressor gene promoters in the presence and absence of transcriptional silencing in high hyperdiploid acute lymphoblastic leukaemia. Br J Haematol.

[57] Lafage-Pochitaloff M, Gerby B, Baccini V, Largeaud L, Fregona V, Prade N (2022). The CADM1 tumor suppressor gene is a major candidate gene in MDS with deletion of the long arm of chromosome 11. Blood Adv.

[58] Kuramochi M, Fukuhara H, Nobukuni T, Kanbe T, Maruyama T, Ghosh HP (2001). TSLC1 is a tumor-suppressor gene in human non-small-cell lung cancer. Nat Genet.

[59] Allinen M, Peri L, Kujala S, Lahti-Domenici J, Outila K, Karppinen SM (2002). Analysis of 11q21-24 loss of heterozygosity candidate target genes in breast cancer: Indications of TSLC1 promoter hypermethylation. Genes Chromosom Cancer.

[60] Fukuhara H, Kuramochi M, Fukami T, Kasahara K, Furuhata M, Nobukuni T (2002). Promoter methylation of TSLC1 and tumor suppression by its gene product in human prostate cancer. Jpn J Cancer Res..

[61] Honda T, Tamura G, Waki T, Jin Z, Sato K, Motoyama T (2002). Hypermethylation of the TSLC1 gene promoter in primary gastric cancers and gastric cancer cell lines. Japanese. J Cancer Res.

[62] Hui ABY, Lo KW, Kwong J, Lam ECW, Chan SYY, Chow LSN (2003). Epigenetic Inactivation of TSLC1 Gene in Nasopharyngeal Carcinoma. Mol Carcinog.

[63] Ito T, Shimada Y, Hashimoto Y, Kaganoi J, Kan T, Watanabe G (2003). Involvement of TSLC1 in progression of esophageal squamous cell carcinoma. Cancer Res.

[64] Jansen M, Fukushima N, Rosty C, Walter K, Altink R, Van Heek T (2002). Aberrant methylation of the 5′ CpG island of TSLC1 is common in pancreatic ductal adenocarcinoma and is first manifest in high-grade PanINs. Cancer Biol Ther.

[65] Lung HL, Cheng Y, Kumaran MK, Liu ETB, Murakami Y, Chan CY (2004). Fine mapping of the 11q22-23 tumor suppressive region and involvement of TSLC1 in nasopharyngeal carcinoma. Int J Cancer.

[66] Steenbergen RDM, Kramer D, Braakhuis BJM, Stern PL, Verheijen RHM, Meijer CJLM (2004). TSLC1 gene silencing in cervical cancer cell lines and cervical neoplasia. J Natl Cancer Inst.

[67] Surace EI, Lusis E, Murakami Y, Scheithauer BW, Perry A, Gutmann DH (2004). Loss of tumor suppressor in lung cancer-1 (TSLC1) expression in meningioma correlates with increased malignancy grade and reduced patient survival. J Neuropathol Exp Neurol.

[68] Fukami T, Fukuhara H, Kuramochi M, Maruyama T, Isogai K, Sakamoto M (2003). Promoter methylation of the TSLC1 gene in advanced lung tumors and various cancer cell lines. Int J Cancer.

[69] Goto A, Niki T, Chi-Pin L, Matsubara D, Murakami Y, Funata N (2005). Loss of TSLC1 expression in lung adenocarcinoma: Relationships with histological subtypes, sex and prognostic significance. Cancer Sci.

[70] Uchino K, Ito A, Wakayama T, Koma YI, Okada T, Ohbayashi C (2003). Clinical implication and prognostic significance of the tumor suppressor TSLC1 gene detected in adenocarcinoma of the lung. Cancer.

[71] Ito A, Okada M, Uchino K, Wakayama T, Koma YI, Iseki S (2003). Expression of the TSLC1 adhesion molecule in pulmonary epithelium and its down-regulation in pulmonary adenocarcinoma other than bronchioloalveolar carcinoma. Lab Investig.

[72] Chijioke O, Müller A, Feederle R, Barros MHM, Krieg C, Emmel V (2013). Human natural killer cells prevent infectious mononucleosis features by targeting lytic epstein-barr virus infection. Cell Rep.

[73] Soneson C, Love MI, Robinson MD (2016). Differential analyses for RNA-seq: Transcript-level estimates improve gene-level inferences. F1000Research.

[74] Patro R, Duggal G, Love MI, Irizarry RA, Kingsford C (2017). Salmon provides fast and bias-aware quantification of transcript expression. Nat Methods.

[75] Love MI, Huber W, Anders S (2014). Moderated estimation of fold change and dispersion for RNA-seq data with DESeq2. Genome Biol.

[76] Strimmer K (2008). fdrtool: A versatile R package for estimating local and tail area-based false discovery rates. Bioinformatics.

[77] Anders S, Huber W (2010). Differential expression analysis for sequence count data. Genome Biol.

[78] Vasilikos L, Hänggi K, Spilgies LM, Kisele S, Rufli S, Wong WWL (2021). Loss of cIAP1 in endothelial cells limits metastatic extravasation through tumor-derived lymphotoxin alpha. Cancers.

[79] Tierney RJ, Shannon-Lowe CD, Fitzsimmons L, Bell AI, Rowe M (2015). Unexpected patterns of Epstein-Barr virus transcription revealed by a High throughput PCR array for absolute quantification of viral mRNA. Virol.

[80] Bell AI, Groves K, Kelly GL, Croom-Carter D, Hui E, Chan ATC (2006). Analysis of Epstein-Barr virus latent gene expression in endemic Burkitt’s lymphoma and nasopharyngeal carcinoma tumour cells by using quantitative real-time PCR assays. J Gen Virol.

