# Supplementary material for: Epstein Barr virus-mediated transformation of B cells from XIAP-deficient patients leads to increased expression of the tumor suppressor CADM1
Source: Cell Death Dis. 2022 Oct 22;13(10):892. doi: 10.1038/s41419-022-05337-z (PMC9587222; doi:10.1038/s41419-022-05337-z)
Supplement: Supplementary file 1 — supplemental figure legends [file 41419_2022_5337_MOESM1_ESM.docx]

**Epstein Barr virus-mediated transformation of B cells from XIAP-deficient patients leads to increased expression of the tumor suppressor CADM1**

Christine Engelmann, Patrick Schuhmachers, Hana Zdimerova, Sanamjeet Virdi, Mathias Hauri-Hohl, Jana Pachlopnik-Schmid, Adam Grundhoff, Rebecca A. Marsh, Wendy Wei-Lynn Wong and Christian Münz

**SUPPLEMENTAL FIGURE LEGENDS**

**Figure S1. relates to Fig. 1**

**A** Total numbers of human CD3^+^, CD4^+^ and CD8^+^ T cells and NKp46^+^ NK cells during the treatment with Birinapant (blue, n = 6) or vehicle (black, n = 5) of humanized mice. The data come from mice reconstituted with hCD34^+^ cells derived from 1 donor. **B** Reduction of cIAP1 in the spleen in Birinapant-treated mice as determined by Western blotting. β-actin served as loading control. Quantification of cIAP1 relative to β-actin. The uncropped Western blots are shown in Figure S7. **C** Total numbers of human CD3^+^, CD4^+^ and CD8^+^ T cells and NKp46^+^ NK cells during the treatment with GT13072 (purple, n = 6) or vehicle (black, n = 4). The data derive from mice reconstituted with hCD34^+^ cells derived from 2 donors. **D** Reduction of cIAP1 in the lung in Birinapant-treated mice as determined by Western blotting. β-actin served as loading control. Quantification of cIAP1 relative to β-actin. **E** XIAP inhibition by GT13072 as shown by TNF-α secretion in PBMC-derived monocytes stimulated with 200 ng/ml L18-MDP. Buffy-coat derived PBMCs were cultured overnight and adherent cells were treated with GT13072 at the indicated concentrations for 30 minutes. L18-MDP (200 ng/ml) was added in the presence of Brefeldin A for 2.5 hours. Data derived from two different donors. **F** Total numbers of human CD4^+^ T cells and NKp46^+^ cells during EBV infection under SMAC-mimetic treatment with Birinapant (blue, n = 8), GT13072 (purple, n = 10) or vehicle (black, n = 11). The data derive from mice reconstituted with hCD34^+^ cells derived from 3 different donors. **G** EBV viral loads in serum of mice from which sufficient volume of blood could be collected at termination (Birinapant, n = 8; GT13072, n = 10; vehicle, n = 7). **H** Total IgM levels in the serum at termination were determined by ELISA. **I** Representative flow cytometry plot of B cell subsets in splenocytes from a PBS and EBV-infected mouse. Frequency of switched memory B cells in PBS mice and EBV infected mice with and without GT13072 treatment. (**A-E**) Each dot represents one animal (median + IQR). Statistical significance was determined by Mann-Whitney *U* test, *p < 0.05; ns, not-significant.

**Figure S2. relates to Fig. 2**

Frequency and total numbers of CD4^+^ (**A**) and CD8^+^ (**B**) T cell subsets, including naïve (CD45RA^+^, CD62L^+^), central memory (Tcm; CD45RA^-^, CD62L^+^), effector memory (Tem; CD45RA^-^, CD62L^-^) and effector memory re-expressing CD45RA (Temra; CD45RA^+^, CD62L^-^) as determined by flow cytometry in healthy donors (HD, black), including 2 children aged 6 and 10 years (white circles), female carriers (M, blue) and XIAP patients (X, red). **C** Representative Western blots of cIAP1 and cIAP2 in FACS-sorted switched-memory and naive B cells. Quantification of cIAP1 and cIAP2 from 2 different donors.

**Figure S3. relates to Fig. 3.**

**A** Normalized read counts of XIAP transcript levels during primary EBV infection of healthy human B cells (source: Mrozek-Gorska et al. 2019; http://ebv-b.helmholtz-muenchen.de/). **B** Total cell count and proliferation rate of individual LCLs. Total cell count as determined by trypan blue exclusion of LCL cell cultures over 4 days (left). Proliferation rate as determined by the inverse of the median fluorescent intensity (MFI) of Cell Trace Violet x10^6^ of each cell line (right). Cells were seeded at 0.3x10^5^ cells / ml after CTV staining and analyzed every day for total cell counts and CTV expression. Data represent the mean with SEM of 2-3 technical replicates.

**Figure S4. relates to Fig. 4.**

**A** RT-qPCR analysis of early lytic and **B** latent EBV genes in healthy donor (C1, C2, C3), mother (M1, M2, M3, M4) and XIAP patient LCLs (X1, X2, X4, X6, X7). Each dot represents one cell line from one subject, derived from 1-3 technical replicates. Statistical significance was determined by Mann-Whitney *U* test with *p < 0.05.

**Figure S5. relates to Fig. 5**

**A** Flow cytometry plots showing the expression of XIAP in four carrier mother (M) LCLs five days after CRISPR/Cas9 knock-out of XIAP (bottom graphs) and mock-knock-out (Cas9 only; top graphs). **B** XIAP expression in mock-knock-out (empty bar) and XIAP-knock-out (gray patterned bar). Each dot represents one cell line. **C** Expression of CADM1 in carrier mother LCLs three weeks after CRISPR/Cas9 control knock-out (empty bars) and in XIAP positive and negative subsets after CRISPR/Cas9 XIAP knock-out (XIAP^+^, XIAP^-^, gray patterned bars). **C** Gene-set enrichment analysis of XIAP-deficient LCLs compared to healthy donor (top) and mother-derived (bottom) LCLs.

**Figure S6. relates to Fig. 5**

**A** CADM1 expression 5 days after ctrl-ko and CADM1-ko in XIAP patients (X1, X2, X4) and healthy donor (HD) LCLs. **B** Total live cell count of ctrl-ko and CADM1-ko cell cultures, as determined by trypan blue exclusion. **C** Gating strategy for CTV an Ki67 staining of ctrl-ko (left) and CADM1-ko (right) cells. **D** Percentage of Ki67^+^ LCLs 5 days after seeding 0.5x10^6^ cells. **E** Proliferation index of CADM1-ko and ctrl-ko LCLs as determined by 1 / of MFI of CTV. **F** Frequency of apoptotic cells in control ctrl-ko cells and CADM1-ko cells after culturing them for 5 days. **G** Killing efficiency of wildtype (WT) and XIAP-ko LCLs by HLA-matched EBNA1-specific T cell clones after 18 hours. Effector target ratio (E:T = 5:1). **H** Degranulation and cytokine secretion after 6 hours in EBNA1-specific T cell clones targeting HLA-matched WT or XIAP-ko LCLs. (**G, H**) Dots represent results from 5-6 different T cell clones performed in duplicates in two separate experiments. Statistical significance was assessed with the Mann-Whitney *U* test.

**Figure S7.** The uncropped Western blots.

**SUPPLEMENTAL INFORMATION**

**Table S1.**

Characteristics of four unrelated XIAP patients (X1-4) and their mothers (M1-4) described in this study, related to Figure 2. The indicated age of each individual corresponds to their respective age when blood was withdrawn. All four patients are monitored and treated at the University Children’s Hospital in Zurich, Switzerland.

**Table S2.**

List of LCLs used in this study for RNAseq analysis. XIAP protein expression is provided as determined by Western blot using α-XIAP antibody clone 48 from BD. LCLs from X1, X2, M1, M2, M3 were newly generated during this study. LCLs C1, C2, C3 were generated by McHugh et al. 2017 and LCLs X5, X6, X7 were generated in the lab of R. Marsh. Reference to the originally described patients is provided for X5, X6 and X7.

**Table S3.**

Quantitative RT-PCR primers used in this study to detect EBV and cellular gene transcripts.

**Table S4.**

crRNAs used in this study to mutate XIAP or CADM1 in LCLs.

**Table S5.**

Antibodies used in this study.

**Data S1.**

RNAseq read counts related to Figures 4 and 5. The left column depicts total read counts of each cell line, combined from three technical replicates, each at ~20 million reads. Right columns show read counts that were mapped to the human genome (Hg38) or EBV genome (NCBI: NC_007605.1).

**Data S2.**

Normalized EBV read counts and statistics related to Figure 4. RNAseq coverage of EBV genes (NCBI: NC_007605.1) in LCLs derived from three healthy donors (HD), three carrier mothers (M) and five XIAP patients (X). Columns on the right show statistical significance and fold change of each gene comparing healthy donors to patients (HD vs X) and carrier mothers to patients (M vs X). P value was determined using a two-tailed Student’s t-test.

**Data S3.**

Differential host gene expression, related to Figure 5. Host gene expression (assembly Hg38) in LCLs derived from three healthy donors versus five XIAP patients.

Host gene expression (assembly Hg38) in LCLs derived from three carrier mothers and five XIAP patients.
