# Supplementary figures and images for: Epstein Barr virus-mediated transformation of B cells from XIAP-deficient patients leads to increased expression of the tumor suppressor CADM1
Source: Cell Death Dis. 2022 Oct 22;13(10):892. doi: 10.1038/s41419-022-05337-z (PMC9587222; doi:10.1038/s41419-022-05337-z)

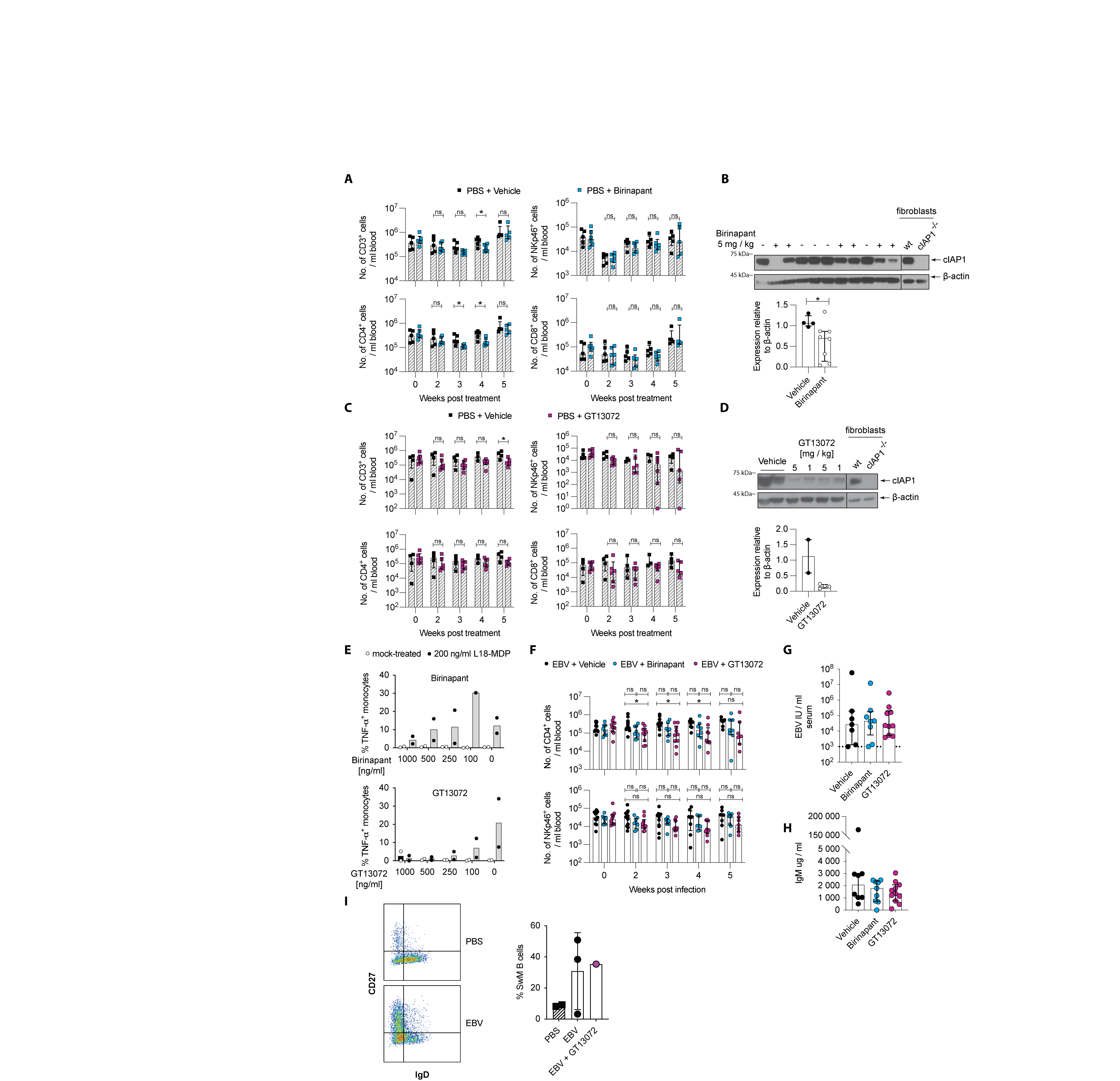

Supplement: Supplementary file 2 — supplemental Figure 1 [file 41419_2022_5337_MOESM2_ESM.png]

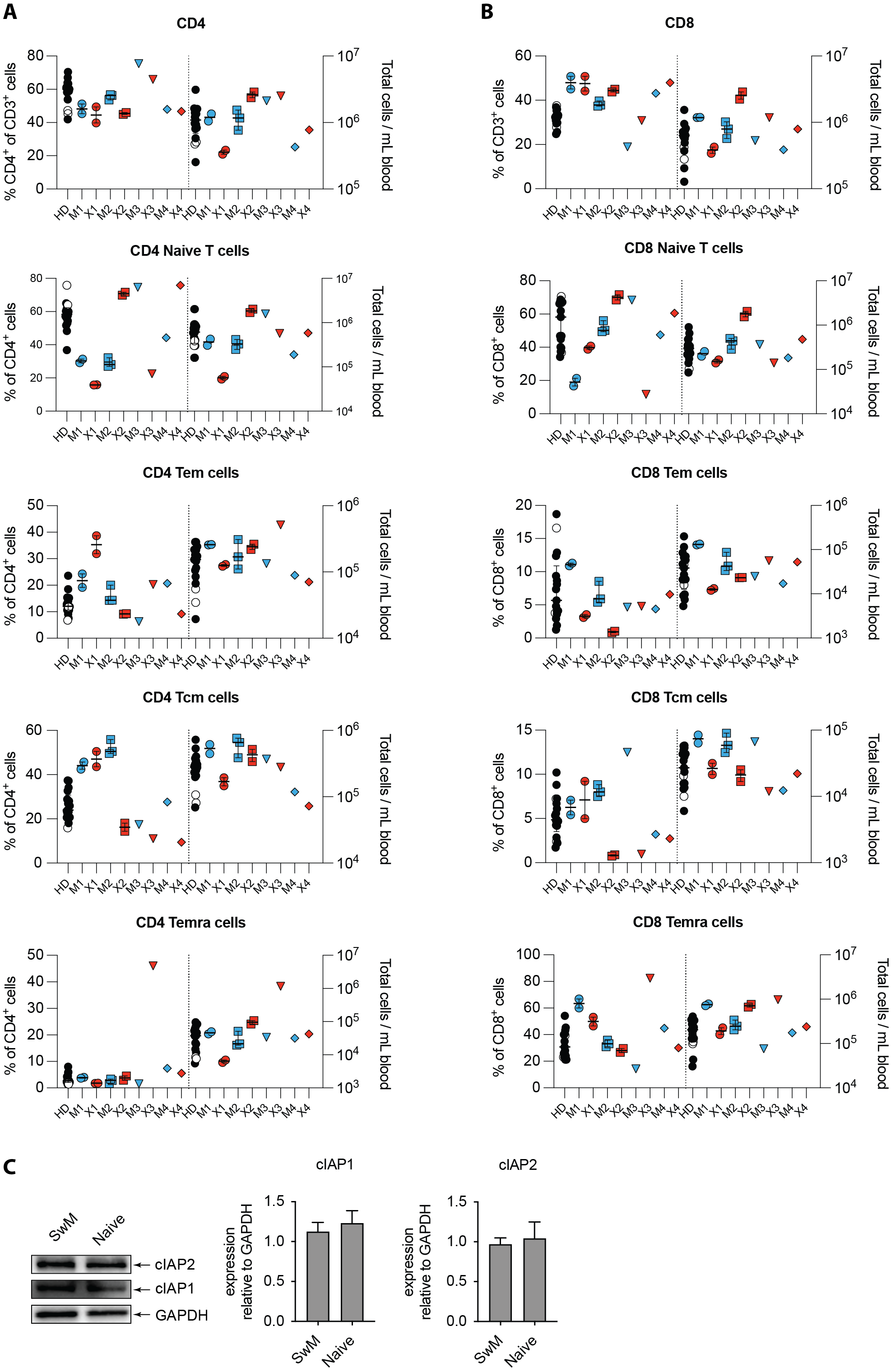

Supplement: Supplementary file 3 — supplemental Figure S2 [file 41419_2022_5337_MOESM3_ESM.png]

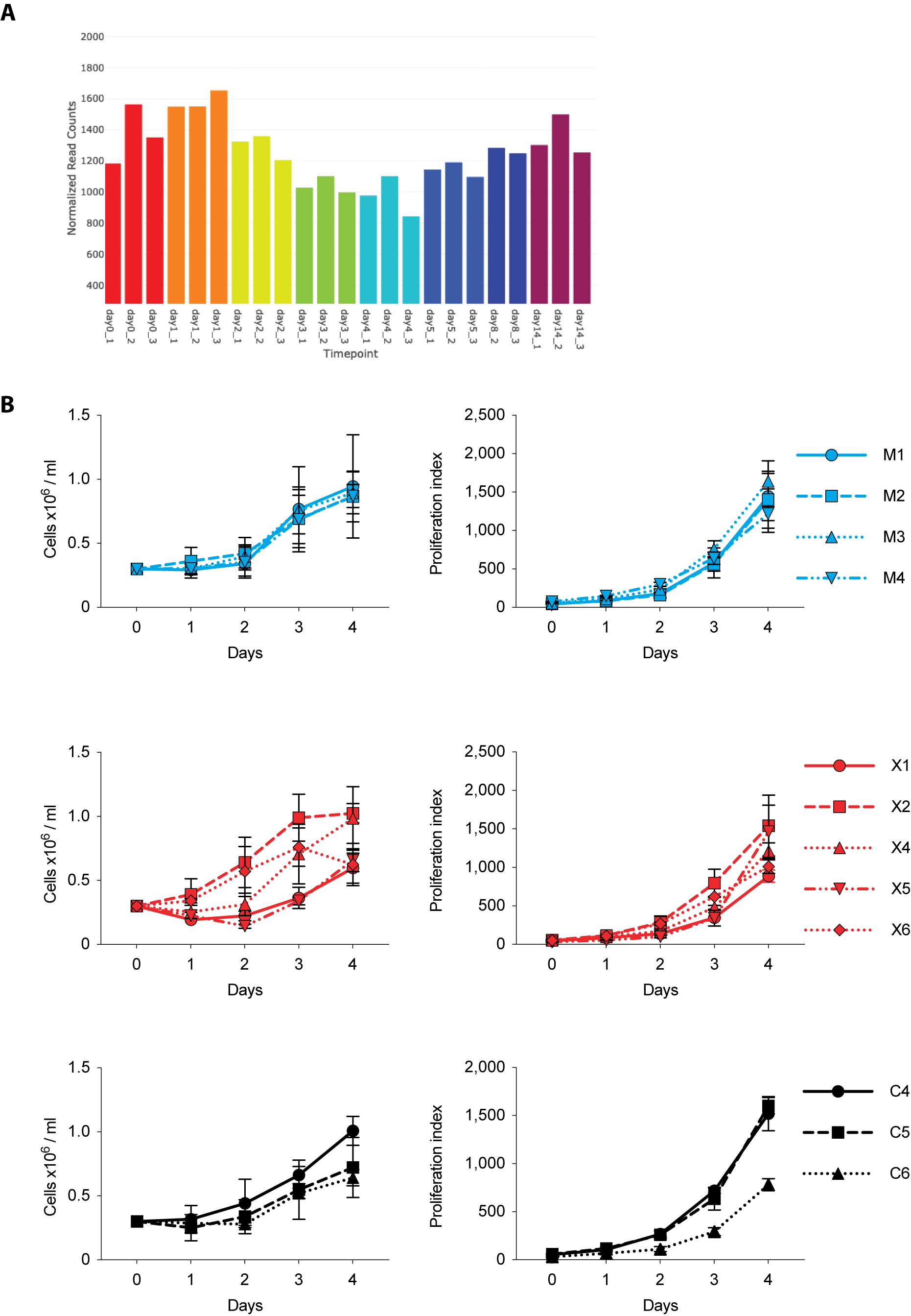

Supplement: Supplementary file 4 — supplemental Figure S3 [file 41419_2022_5337_MOESM4_ESM.png]

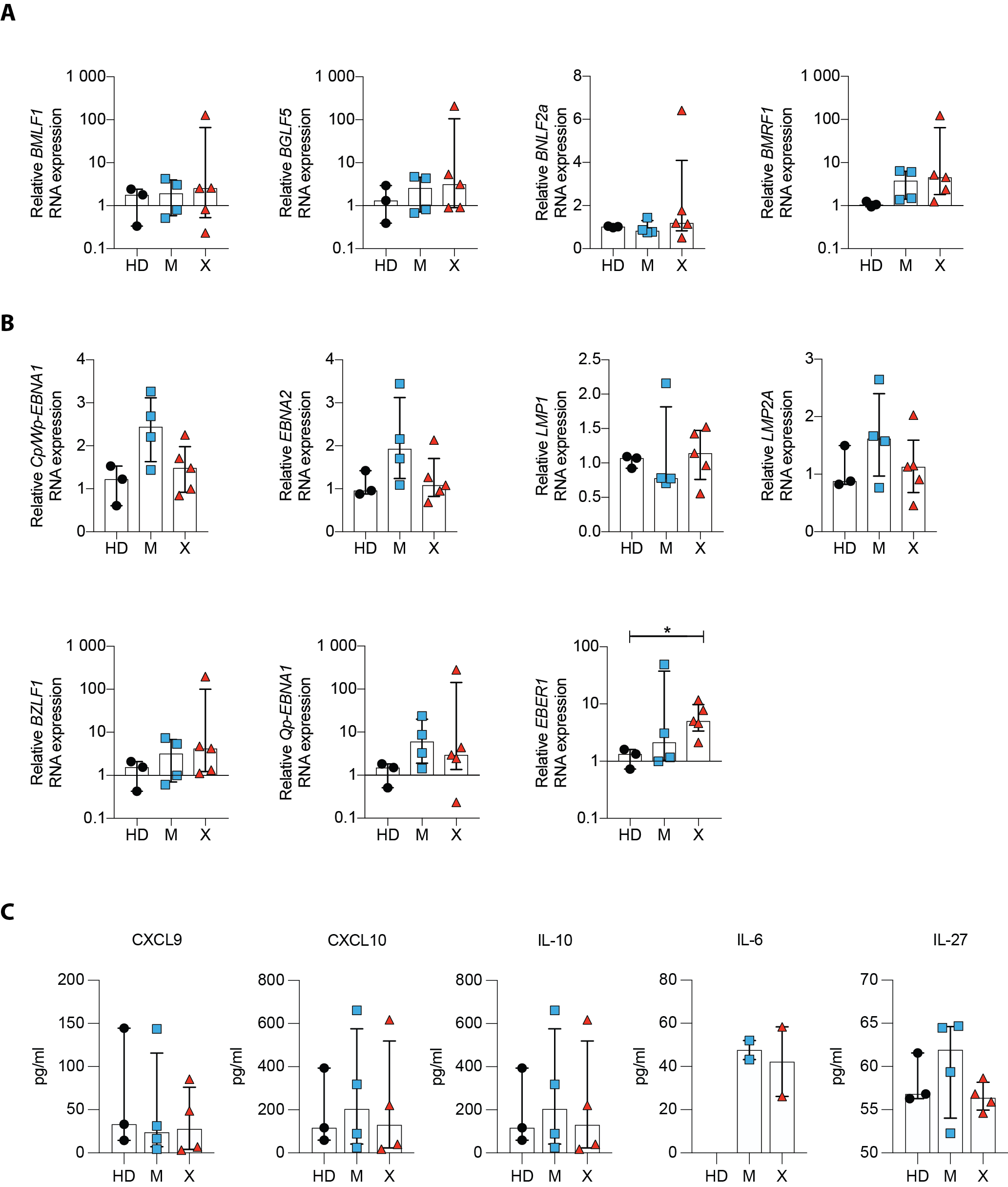

Supplement: Supplementary file 5 — supplemental Figure S4 [file 41419_2022_5337_MOESM5_ESM.png]

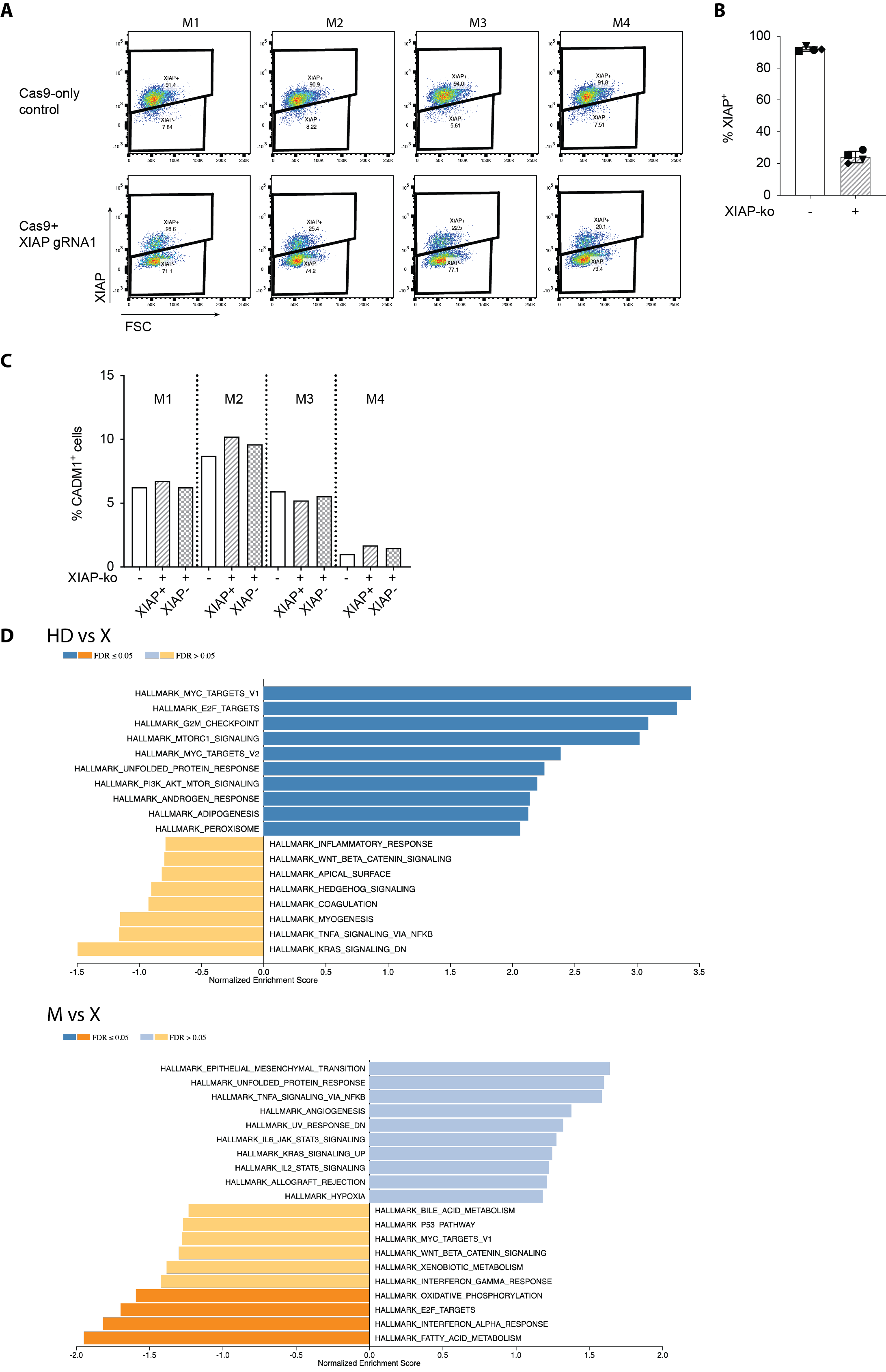

Supplement: Supplementary file 6 — supplemental Figure S5 [file 41419_2022_5337_MOESM6_ESM.png]

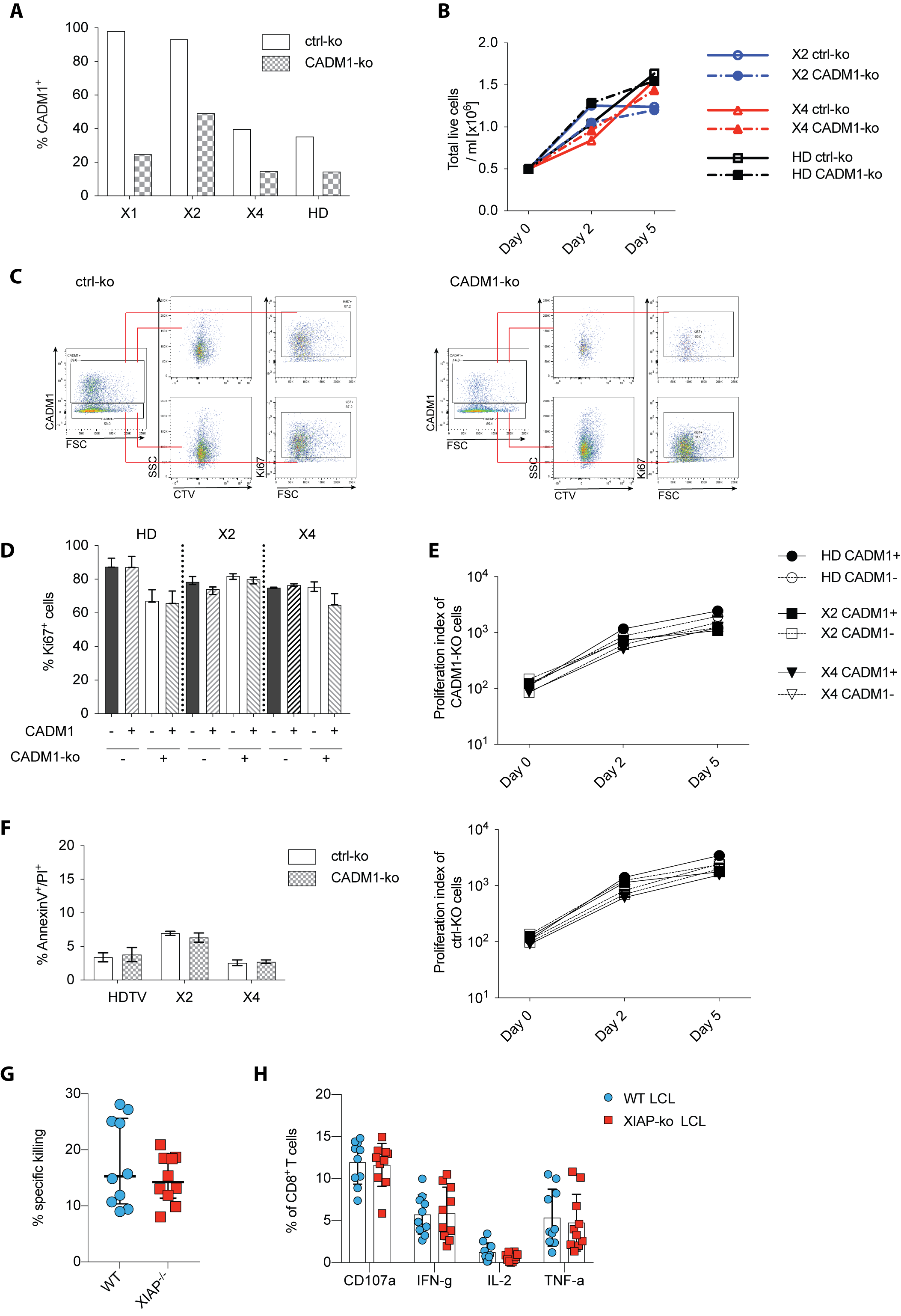

Supplement: Supplementary file 7 — supplemental Figure S6 [file 41419_2022_5337_MOESM7_ESM.png]
