# Supplementary material for: Epstein Barr virus-mediated transformation of B cells from XIAP-deficient patients leads to increased expression of the tumor suppressor CADM1
Source: Cell Death Dis. 2022 Oct 22;13(10):892. doi: 10.1038/s41419-022-05337-z (PMC9587222; doi:10.1038/s41419-022-05337-z)
Supplement: Supplementary file 16 — Original Data File [file 41419_2022_5337_MOESM16_ESM.pptx]

## Slide 1
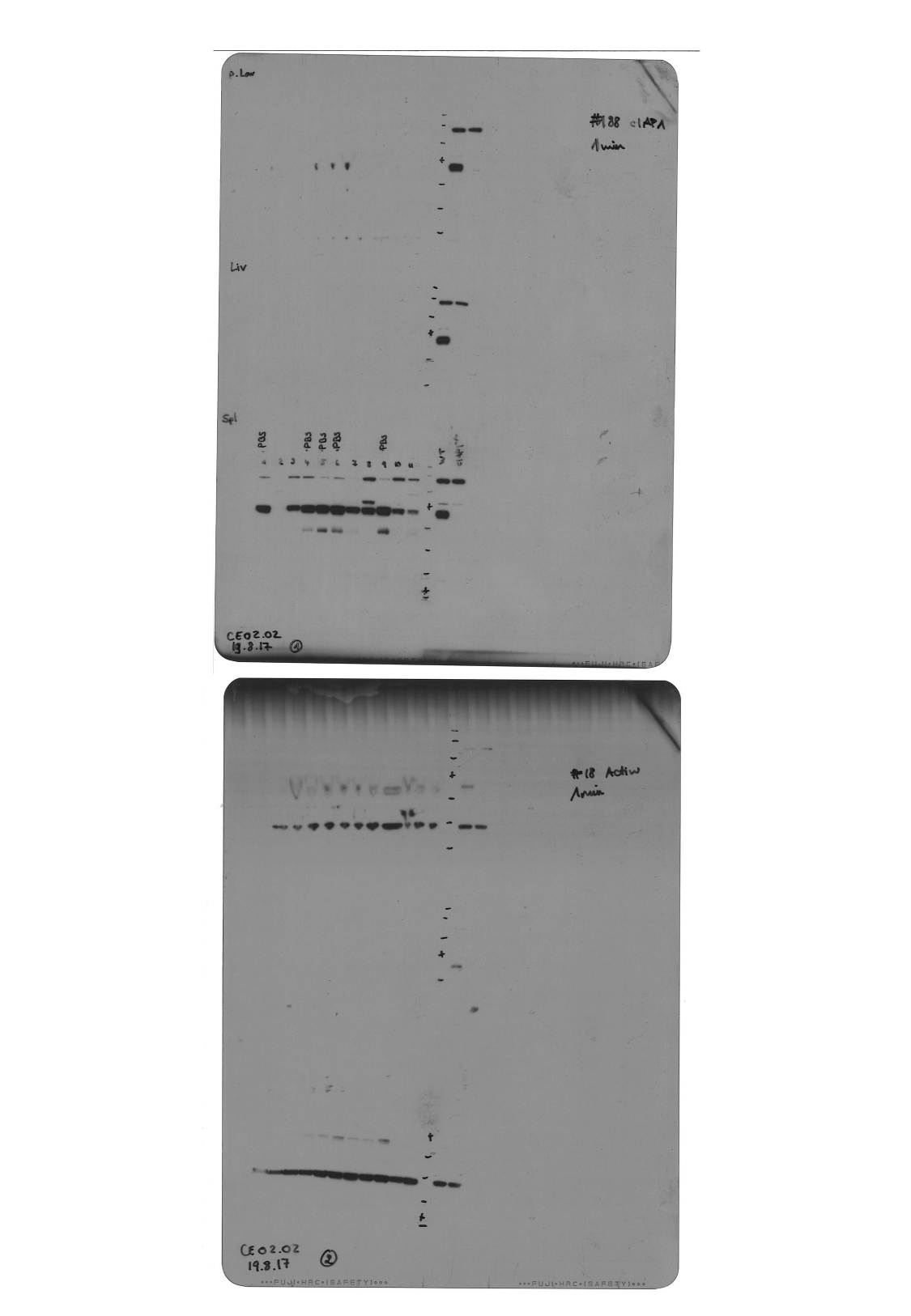

## Slide 2
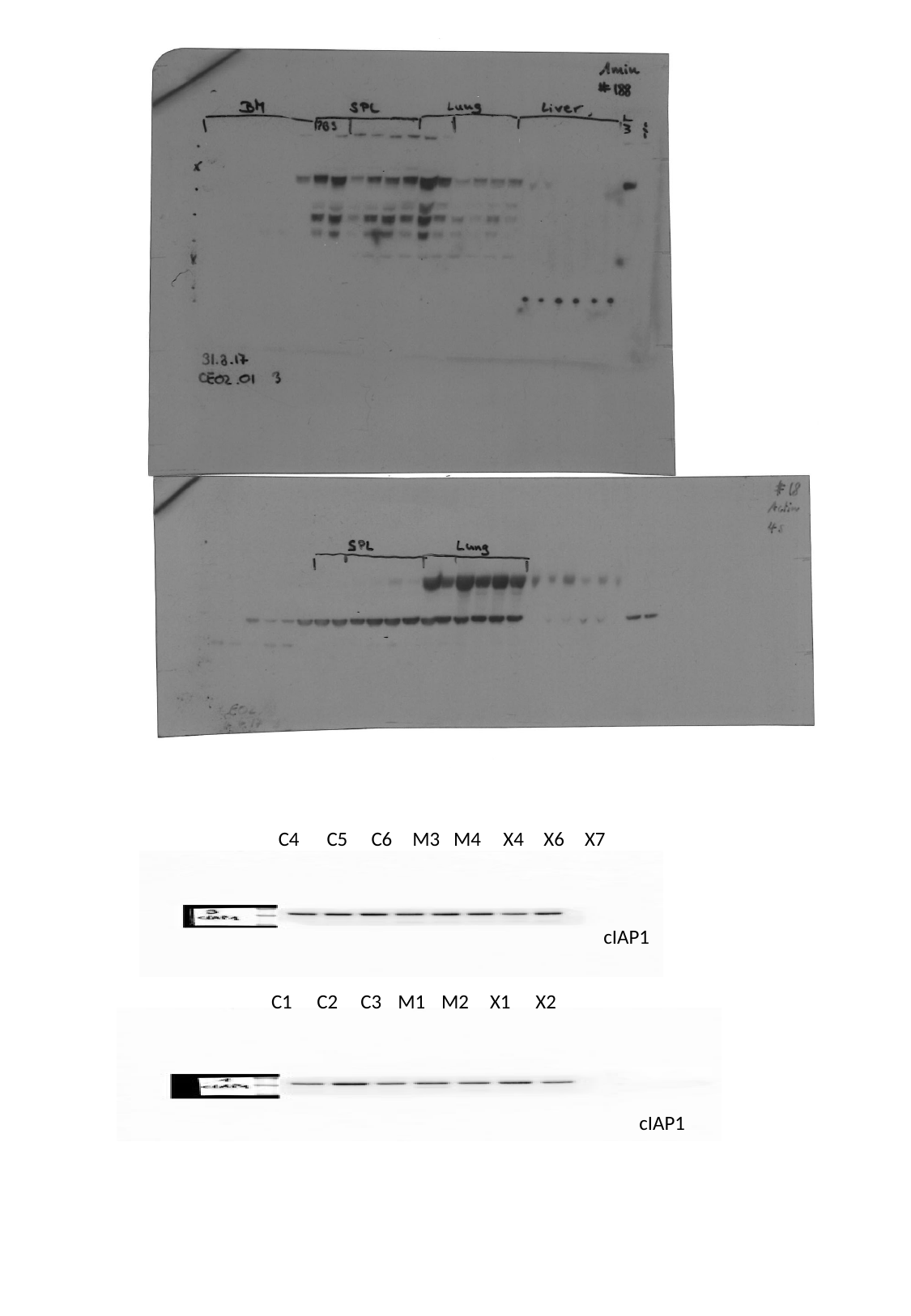

C4
C5
C6
M3
M4
X4
X6
X7
cIAP1
C1
C2
C3
M1
M2
X1
X2
cIAP1

## Slide 3
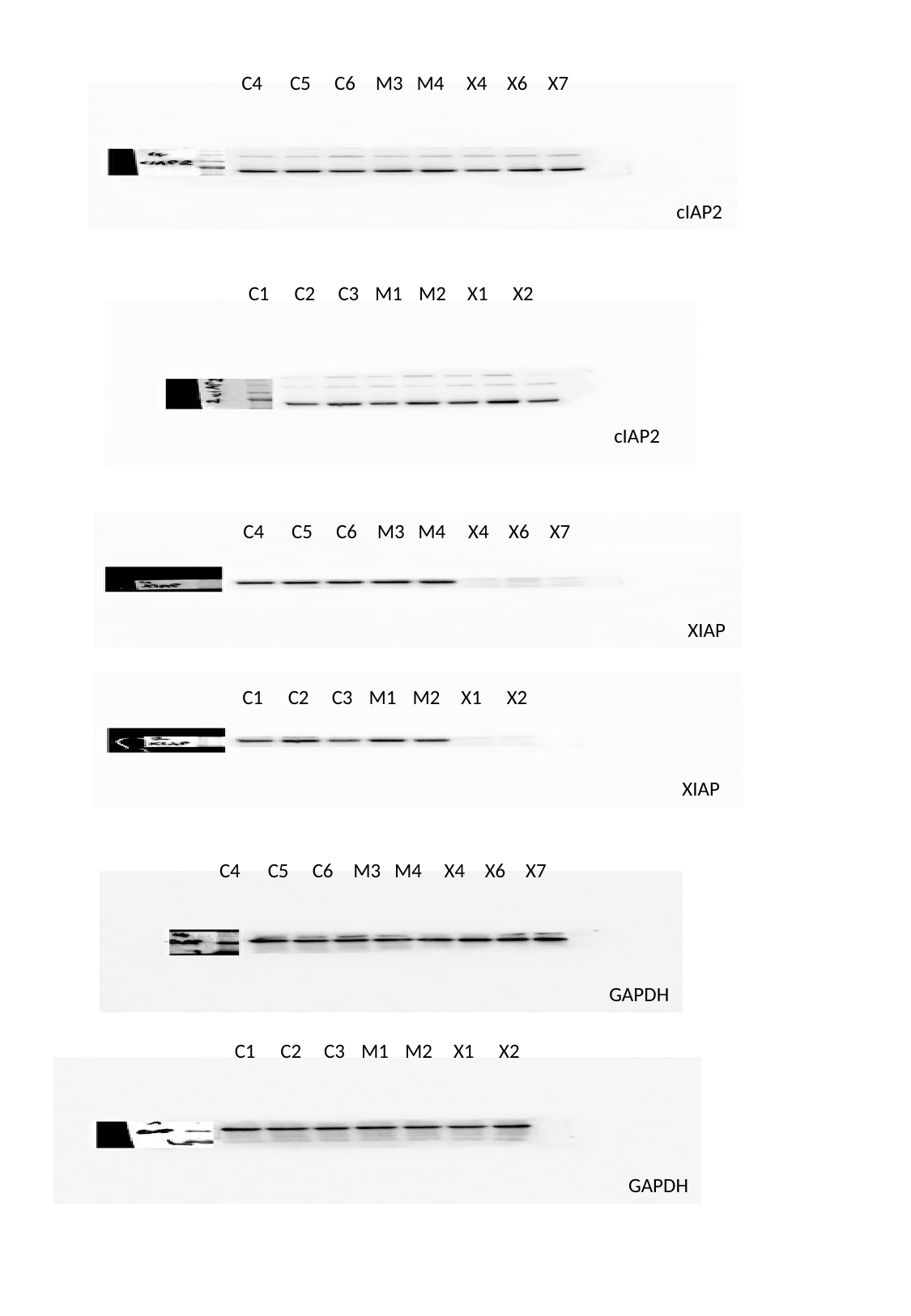

C4
C5
C6
M3
M4
X4
X6
X7
cIAP2
C1
C2
C3
M1
M2
X1
X2
cIAP2
C4
C5
C6
M3
M4
X4
X6
X7
XIAP
C1
C2
C3
M1
M2
X1
X2
XIAP
C4
C5
C6
M3
M4
X4
X6
X7
GAPDH
C1
C2
C3
M1
M2
X1
X2
GAPDH

## Slide 4
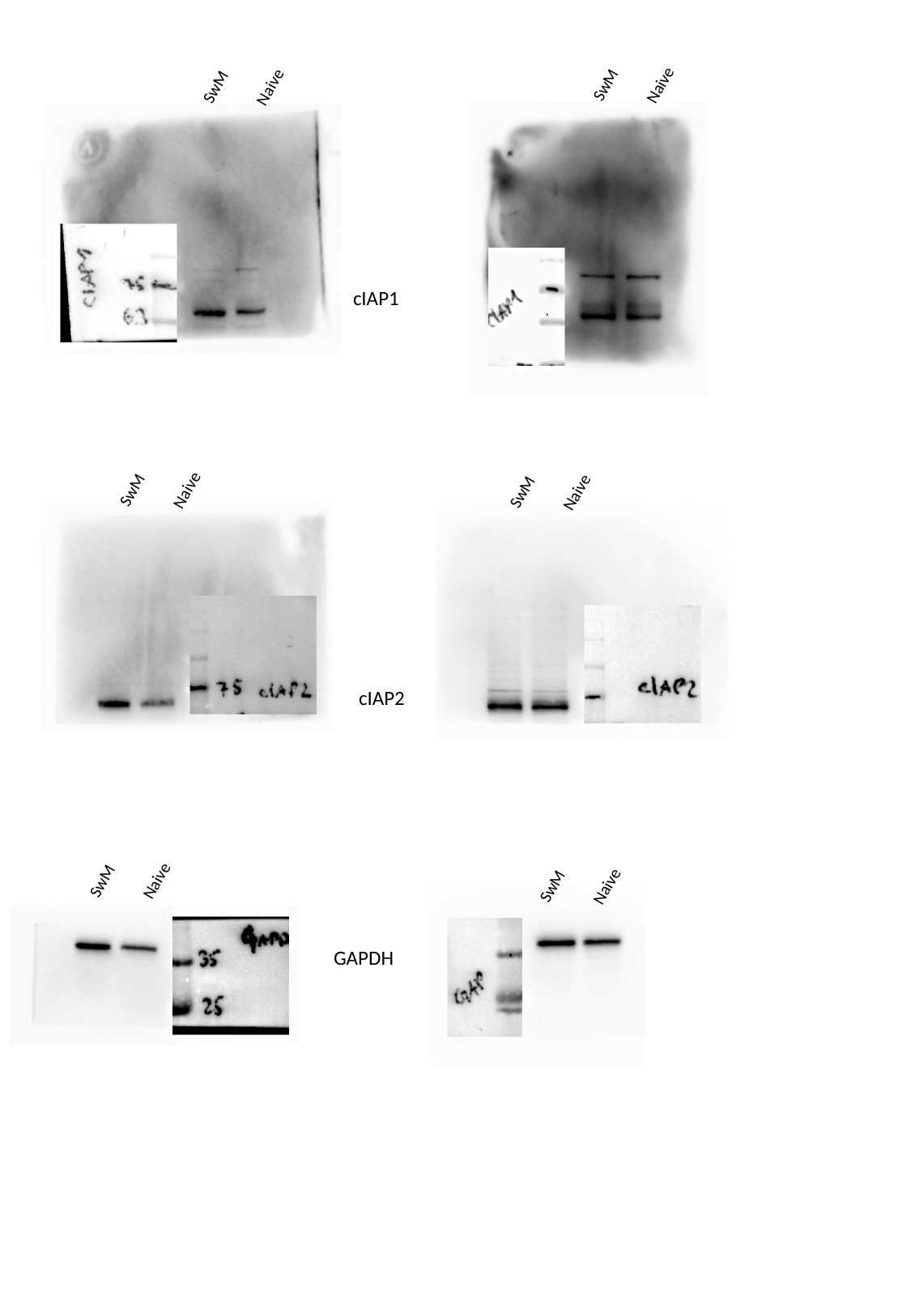

SwM
Naive
SwM
Naive
cIAP1
SwM
Naive
SwM
Naive
cIAP2
SwM
Naive
SwM
Naive
GAPDH
